# Electrically driven amplified spontaneous emission from colloidal quantum dots

**DOI:** 10.1038/s41586-023-05855-6

**Published:** 2023-05-03

**Authors:** Namyoung Ahn, Clément Livache, Valerio Pinchetti, Heeyoung Jung, Ho Jin, Donghyo Hahm, Young-Shin Park, Victor I. Klimov

**Affiliations:** 1 Nanotechnology and Advanced Spectroscopy Team, C-PCS, Chemistry Division, Los Alamos National Laboratory, Los Alamos, NM USA; 2 Center for High Technology Materials, University of New Mexico, Albuquerque, NM USA

**Keywords:** Diode lasers, Quantum dots

## Abstract

Colloidal quantum dots (QDs) are attractive materials for realizing solution-processable laser diodes that could benefit from size-controlled emission wavelengths, low optical-gain thresholds and ease of integration with photonic and electronic circuits^1–7^. However, the implementation of such devices has been hampered by fast Auger recombination of gain-active multicarrier states^1,8^, poor stability of QD films at high current densities^9,10^ and the difficulty to obtain net optical gain in a complex device stack wherein a thin electroluminescent QD layer is combined with optically lossy charge-conducting layers^11–13^. Here we resolve these challenges and achieve amplified spontaneous emission (ASE) from electrically pumped colloidal QDs. The developed devices use compact, continuously graded QDs with suppressed Auger recombination incorporated into a pulsed, high-current-density charge-injection structure supplemented by a low-loss photonic waveguide. These colloidal QD ASE diodes exhibit strong, broadband optical gain and demonstrate bright edge emission with instantaneous power of up to 170 μW.

## Abstract (web summary)

Colloidal quantum dot devices demonstrating electrically pumped amplified spontaneous emission are described, showing strong, broadband optical gain and bright edge emission, opening the path to solution-processable laser diodes.

## Main

Electrically pumped lasers or laser diodes based on solution-processable materials have long been desired devices for their compatibility with virtually any substrate, scalability and ease of integration with on-chip photonics and electronics. Such devices have been pursued across a wide range of materials, including polymers^14–16^, small molecules^17,18^, perovskites^19,20^ and colloidal QDs^1–7^. The last materials are especially attractive for implementing laser diodes because, as well as being compatible with inexpensive and easily scalable chemical techniques, they offer several advantages derived from a zero-dimensional character of their electronic states^21,22^. These include a size-tunable emission wavelength, low optical-gain thresholds and high temperature stability of lasing characteristics stemming from a wide separation between their atomic-like energy levels^21–23^.

Several challenges complicate the realization of colloidal QD laser diodes. These include extremely fast nonradiative Auger recombination of optical-gain-active multicarrier states^1,8^, poor stability of QD solids under high current densities required to achieve lasing^9,10^ and unfavourable balance between optical gain and optical losses in electroluminescent devices wherein a gain-active QD medium is a small fraction of the overall device stack comprising several optically lossy charge-transport layers^11–13^.

Here we resolve these challenges using engineered QDs with suppressed Auger recombination and a special electroluminescent-device architecture, which features a photonic waveguide consisting of a bottom distributed Bragg reflector (DBR) and a top silver (Ag) electrode. The transverse optical cavity formed by the DBR and the Ag mirror improves field confinement in the QD gain medium and simultaneously reduces optical losses in charge-conducting layers. It also facilitates the build-up of ASE owing to improved collection of spontaneous seed photons and the increased propagation path in the QD medium. As a result, we achieve large net optical gain with electrical pumping and demonstrate room-temperature ASE at the band-edge (1S) and excited-state (1P) transitions.

**Fig. 1:**
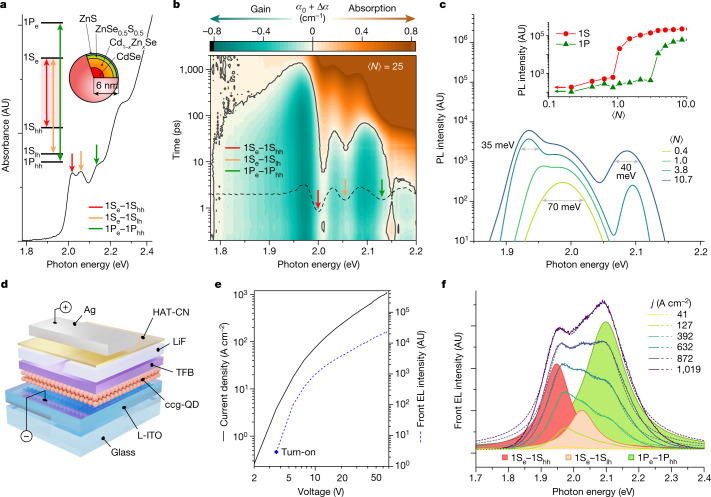
Optical and EL properties of ccg-QDs. **a**, The ground-state absorption spectrum (*α*_0_) of the CdSe/Cd_1−*x*_Zn_*x*_Se/ZnSe_0.5_S_0.5_/ZnS ccg-QDs (top-right inset). Vertical arrows mark the three lowest-energy transitions involving 1S and 1P electron and hole states (shown in the top-left inset). **b**, Transient absorption (TA) measurements of the ccg-QD solution sample conducted using 110-fs, 2.4-eV pump pulses with per-pulse fluence *w*_p_ = 1.6 mJ cm^−2^ (⟨*N*⟩ = 25). The TA signal is presented as *α*(*hv*,*t*) = *α*_0_(*hv*) + Δ*α*(*hv*,*t*), where *α*_0_ and *α* are the absorption coefficients of the unexcited and excited sample, respectively, and Δ*α* is the pump-induced absorption change. The solid black line (*α* = 0) separates the regions of absorption (*α* > 0, brown) and optical gain (*α* < 0; green). The dashed black line is the second derivative of *α*_0_ (panel **a**). **c**, Pump-intensity-dependent spectra of edge-emitted photoluminescence (PL) of a 300-nm-thick ccg-QD film on a glass substrate under excitation with 110-fs, 3.6-eV pump pulses. The pump spot is shaped as a narrow 1.7-mm-long stripe orthogonal to the sample edge. The emergence of narrow peaks at 1.93 eV and 2.08 eV (full width at half maximum 35 meV and 40 meV, respectively) at higher ⟨*N*⟩ indicates the transition to the ASE regime. On the basis of the onset of sharp intensity growth (inset), the 1S and 1P ASE thresholds are, respectively, about 1 and about 3 excitons per dot on average. **d**, A device stack of the reference LED comprises an L-ITO cathode, a ccg-QD layer and TFB/HAT-CN hole transport/injection layers separated by a LiF spacer with a current-focusing aperture. The device is completed with a Ag anode prepared as a narrow strip. **e**, The *j*–*V* (solid black line) and EL intensity–*V* (dashed blue line) dependences of the reference device. **f**, The *j*-dependent EL spectra of front (surface) emission of the reference device. The EL spectrum recorded at 1,019 A cm^−2^ is deconvolved into three Lorentzian bands that correspond to the three ccg-QD transitions shown in **a**. AU, arbitrary units.

In this study, we use an optical gain medium based on a revised version of continuously graded QDs (cg-QDs), which are similar to our previously introduced CdSe/Cd_1−*x*_Zn_*x*_Se cg-QDs^9^ but feature a reduced thickness of the graded layer. These ‘compact’ cg-QDs (abbreviated as ccg-QDs)^13^ comprise a CdSe core of 2.5 nm radius, a 2.4-nm-thick graded Cd_1−*x*_Zn_*x*_Se layer and a final protective shell made of ZnSe_0.5_S_0.5_ and ZnS layers with 0.9 nm and 0.2 nm thicknesses, respectively (Fig. 1a, top-right inset and Supplementary Fig. 1). Despite its reduced thickness, the compact graded shell allows for highly effective suppression of Auger decay^24^, which leads to a long biexciton Auger lifetime (*τ*_XX,A_ = 1.9 ns) and a correspondingly high biexciton emission quantum yield of 38% (Supplementary Fig. 2). The compact graded shell also produces strong asymmetric compression of the emitting core, which increases the light-heavy hole splitting (*Δ*_lh-hh_) to about 56 meV (ref. ^25^) (Fig. 1a). This impedes thermal depopulation of the band-edge heavy-hole state and thereby reduces the optical gain threshold^7^.

Notably, the reduced shell thickness allows for an increased QD packing density in film samples and, as a result, leads to enhanced optical gain, which spans across the 1S and 1P transitions and exhibits a wide bandwidth of about 420 meV (Fig. 1b). These properties facilitate the development of ASE, which is readily observed for optically excited ccg-QD films (Fig. 1c). The ASE occurs at both the 1S and 1P transitions and exhibits low excitation thresholds ⟨*N*_th,ASE_⟩ ≈ 1 (1S) and 3 (1P) excitons per dot on average. On the basis of the variable stripe length (VSL) ASE measurements of a 300-nm-thick ccg-QD film, the 1S and 1P optical gain coefficients are 780 cm^−1^ and 890 cm^−1^, respectively (Supplementary Fig. 3). Owing to a near-unity mode confinement factor of the measured film, we will refer to the derived values as ‘material gain’ coefficients (*G*_mat,1S_ and *G*_mat,1P_, respectively).

Initially, we incorporate ccg-QDs into ‘reference’ light-emitting diodes (LEDs) whose architecture is similar to that in refs. ^12,13^. These devices (Fig. 1d) are assembled on top of a glass substrate and comprise a ccg-QD active layer (approximately three monolayers thick) sandwiched between a bottom electrode (cathode) made of low-index indium tin oxide (L-ITO) and an organic hole-transport layer (HTL) of poly[(9,9-dioctylfluorenyl-2,7-diyl)-alt(4,4′-(N-(4-butylphenyl)))] (TFB). The L-ITO electrode is made of a mixture of standard ITO and SiO_2_, which reduces optical losses and enhances refractive-index contrast at the QD–cathode interface, thereby improving optical-mode confinement in the QD layer^11^. The TFB HTL is separated from the organic hole-injection layer (HIL) made of dipyrazino[2,3-f:2′,3′-h]quinoxaline-2,3,6,7,10,11-hexacarbonitrile (HAT-CN) by an insulating 50-nm-thick LiF spacer containing a ‘current-focusing’ 30-μm-wide slit^10,12,13^. The device is completed with a silver electrode (anode) prepared as a 300-μm-wide strip orthogonal to the slit in the LiF interlayer. This approach leads to two-dimensional current focusing and allows us to limit the injection area to 30 × 300 μm^2^. The fabricated LEDs, as well as other devices studied in this work, were characterized at room temperature in air.

**Extended Data Fig. 1:**
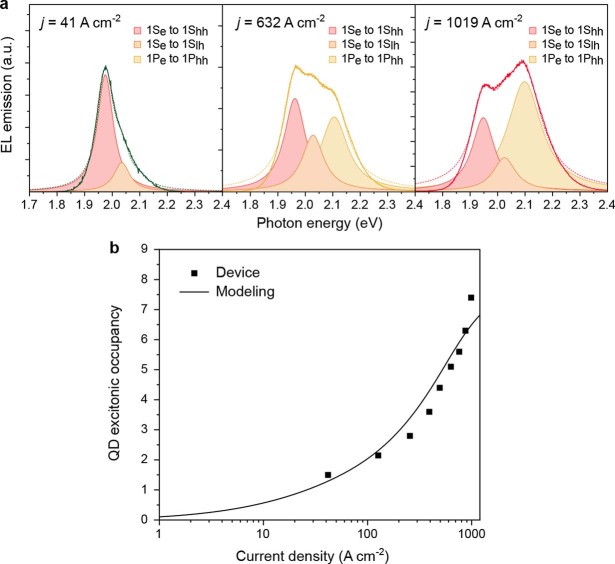
Characterization of the reference electroluminescent device. **a**, The EL spectra of the reference device (Fig. 1f) are deconvolved using three Lorentzian bands that correspond to the 1S_e_–1S_hh_, 1S_e_–1S_lh_ and 1P_e_–1P_hh_ transitions. This procedure is applied to the EL spectra measured at *j* = 41 A cm^−2^ (left), 632 A cm^−2^ (middle) and 1,019 A cm^−2^ (right). **b**, The average QD excitonic occupancy, ⟨*N*⟩, in the active device volume as a function of current density. Symbols are experimental values of ⟨*N*⟩ obtained from the measured ratio of the 1P and 1S EL intensities^10^. The line is calculations conducted using the model of ref. ^10^. According to this analysis, ⟨*N*⟩ reaches 7.4 at *j* = 1,019 A cm^−2^. This is above the gain thresholds for both the 1S and 1P transitions^10,12^. a.u., arbitrary units.

In Fig. 1e,f, we show electroluminescence (EL) measurements of one of the reference LEDs excited using pulsed bias (1-μs pulse duration, 1-kHz repetition rate) with a voltage amplitude (*V*) up to 67 V. At the maximal voltage, the current density (*j*) reaches 1,019 A cm^−2^ (Fig. 1e), which is comparable with values realized with previous current-focusing, pulsed LEDs^10^. The device emission turns on at about 3 V, after which the EL intensity exhibits fast growth. The EL spectra measured at lower *j* peaked at 1.96 eV (1S feature), which corresponds to the band-edge 1S_e_–1S_hh_ transition (Fig. 1f). As *j* is increased, the EL exhibits a pronounced broadening owing to increasing intensities of the higher energy bands associated with the 1S_e_–1S_lh_ (2.02 eV) and the 1P_e_–1P_hh_ (2.1 eV; 1P feature) transitions (Fig. 1f and Extended Data Fig. 1a). At the highest *j*, the EL spectrum peaks at the position of the 1P band, which is indicative of a high per-dot excitonic number realized in these devices. In particular, on the basis of the ratio of the 1P-band and 1S-band amplitudes, the average QD excitonic occupancy ⟨*N*⟩ reaches roughly 7.4 (Extended Data Fig. 1b), which is higher than the optical gain threshold for both the 1S and 1P transitions (Fig. 1c).

**Fig. 2:**
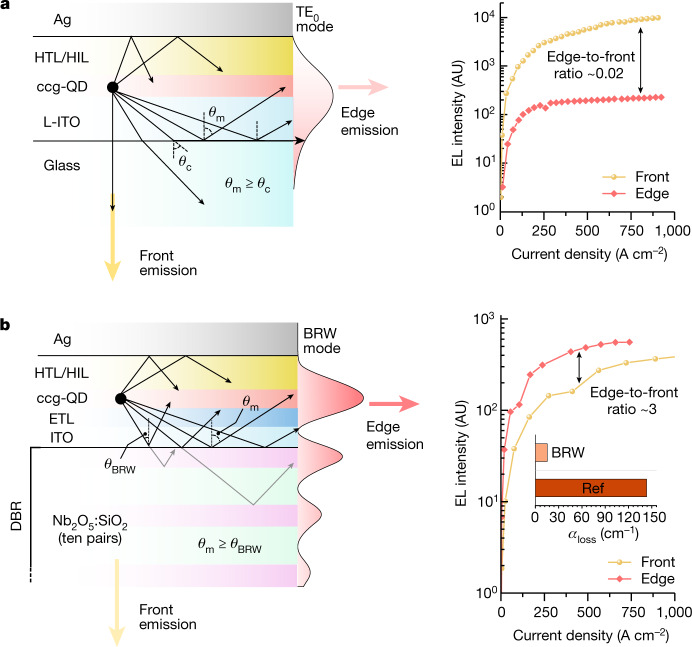
Guided optical modes in the reference and BRW devices. **a**,**b** Left, cross-sectional structure of the reference (**a**) and BRW (**b**) devices, along with the computed distribution of the TE modes (shown as red-shaded profiles). In the reference device, this mode is supported by TIR, whose critical angle (*θ*_c_) is controlled by the refractive-index contrast at the L-ITO–glass interface (sin*θ*_c_ = *n*_glass_/*n*_L-ITO_). In the BRW device, the mode angle (*θ*_m_ = *θ*_BRW_) is defined by the condition of constructive interference (Bragg condition) of reflections from different layers of the DBR. As a result, the optical-field profile exhibits an oscillatory pattern linked to the periodic structure of the DBR. Right, dependence of front-emitted and edge-emitted light intensities (yellow and red symbols, respectively) on current density for the reference (**a**) and BRW (**b**) devices. Owing to large propagation losses, the reference device radiates primarily  from its front glass-cladded surface (the front-to-edge intensity ratio is about 50). By contrast, owing to reduced optical losses (inset in **b**, right) and strong amplification of guided light, the BRW emits more strongly from its edge (the edge-to-front intensity ratio is about 2 to 3). AU, arbitrary units.

Despite achieving population inversion, the reference devices do not exhibit ASE under electrical pumping in either front (surface) or edge emission. This indicates that the overall optical loss overwhelms optical gain generated in a thin QD medium. Photonic modelling of the reference LEDs using a finite element method confirms this assessment (Supplementary Note 1). In these devices, light amplification occurs because of optical modes guided by total internal reflection (TIR) at the L-ITO–glass interface and by the reflection at the silver mirror (Fig. 2a). Because of strong quenching by the metal layer, transverse magnetic (TM) modes experience strong attenuation, therefore, the modes preferred by ASE are of transverse electric (TE) character^12,13^.

**Extended Data Fig. 2:**
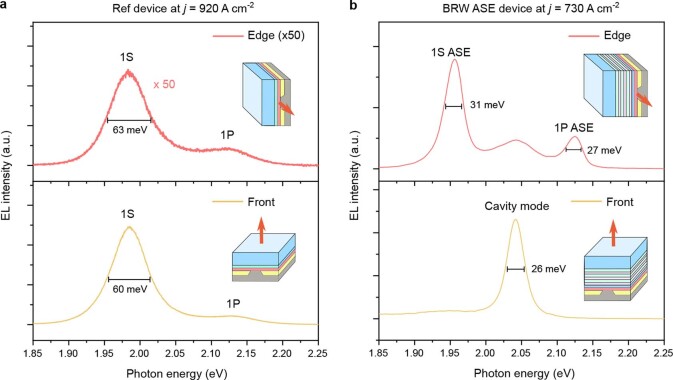
Comparison of edge-emitted and surface-emitted EL spectra of the reference and the BRW devices. **a**, The spectra of edge-emitted and surface-emitted EL (top and bottom subpanels, respectively) of the reference device (insets illustrate the configuration of the measurements) do not show any apparent qualitative distinctions (the device is operated at 920 A cm^−2^). Furthermore, the edge emission is about 50 times weaker than surface emission. **b**, By contrast, the BRW device shows a substantial difference in the spectral shape of edge-emitted and surface-emitted EL (top and bottom subpanels, respectively); *j* = 730 A cm^−2^. Furthermore, edge emission is about two times more intense than surface emission. The spectrum of edge-emitted EL is dominated by 1S and 1P ASE features, whereas the surface emission comprises a single narrow peak at 2.05 eV owing to a vertical Fabry–Pérot cavity formed by the bottom DBR and the top silver mirror. a.u., arbitrary units.

In Fig. 2a, left, we show the computed electric-field distribution of the TE_0_ TIR mode. The mode confinement factor for the QD layer (*Γ*_QD_) is 0.23, which yields the maximal 1S modal gain (*G*_mod,1S_ = *Γ*_QD_*G*_mat,1S_) of about 180 cm^−1^. Notably, a considerable fraction of the optical mode resides in the optically lossy L-ITO electrode. This leads to a large optical loss coefficient (*α*_loss_) of about 140 cm^−1^ (refs. ^12,13^). Although it is slightly lower than *G*_mod,1S_, light absorption in the top Ag electrode and unaccounted light scattering at imperfections within the waveguide increase the overall optical loss such that it becomes greater than modal gain, which suppresses ASE. Because of high propagation losses, the reference device exhibits very weak edge emission and radiates light primarily from the glass-cladded bottom surface such that the ratio of the surface-to-edge emission intensities is about 50 (Fig. 2a, right). Owing to the lack of light amplification, the spectrum of edge emission replicates that of surface EL at all *j* (Extended Data Fig. 2a).

**Extended Data Fig. 3:**
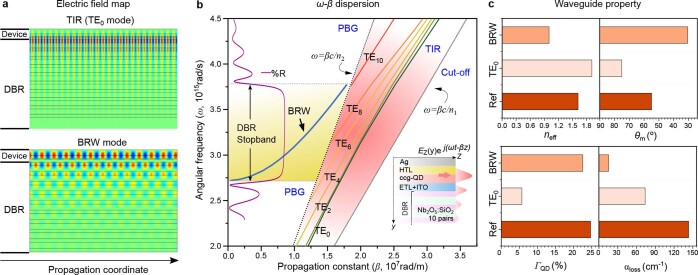
Photonic modelling of the BRW devices developed in the present study. **a**, Contour maps of a TE field of the fundamental TIR mode (top) and the BRW mode (bottom) supported by the structure with the transverse DBR-Ag cavity. The optical field of the BRW mode is confined primarily in the device layer (waveguide core), whereas the TIR mode concentrates at the device–DBR interface and leaks into the DBR. **b**, The calculated *ω–β* dispersion (*ω* is the photon angular frequency and *β* is the modulus of the wavevector) of the TE modes allowed in the BRW structure (depicted in the inset). In this case, the highest (*n*_2_) and lowest (*n*_1_) index materials of the waveguide are Nb_2_O_5_ and SiO_2_, respectively. There are no waveguided modes for *n*_eff_ (= *βc*/*ω*) > *n*_2_, which corresponds to the ‘cut-off’ regime. In the range *n*_1_ < *n*_eff_ < *n*_2_ (red-shaded area), several TIR modes are supported by the waveguide owing to reflections from various layers of the thick DBR stack. The range *n*_eff_ < *n*_1_ corresponds to a photonic bandgap or a stopband defined by the reflection spectrum of the DBR (purple line). A BRW mode (blue line) is located in the stopband of the photonic structure. **c**, A comparison of guided mode parameters between the TE_0_ TIR (pink) and BRW (orange) modes of the DBR-based structure (Fig. 2b) and the TE_0_ TIR mode (red) of the reference device (Fig. 2a). The calculated parameters include the effective refractive indices (*n*_eff_), the modal angles (*θ*_m_), the mode confinement factors for the ccg-QD layer (*Γ*_QD_) and the optical-loss coefficients (*α*_loss_).

**Extended Data Fig. 4:**
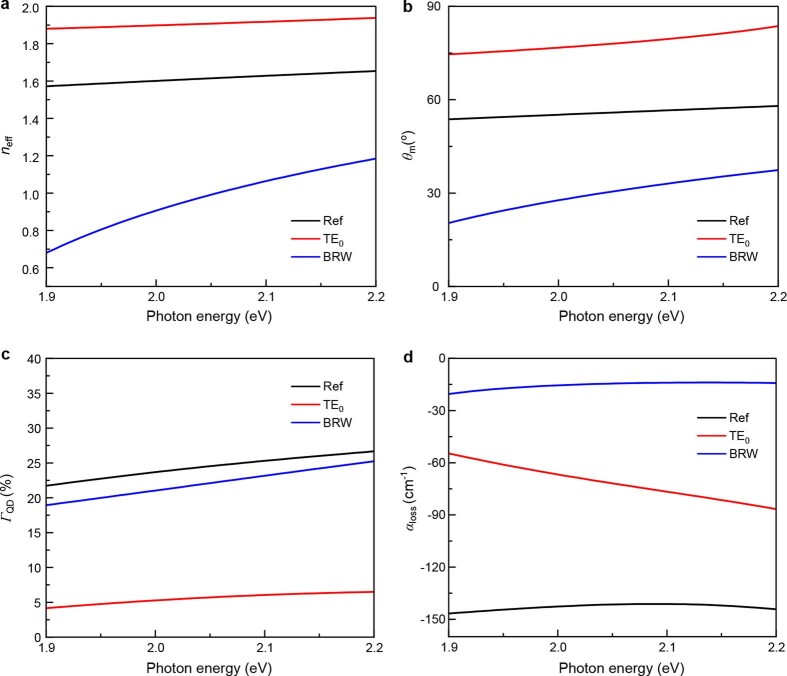
The calculated mode parameters as a function of photon energy. The spectral dependences of the effective refractive index (**a**), the modal angle (**b**), the mode confinement factor for the ccg-QD layer (**c**) and the optical-loss coefficient (**d**) for the reference device (denoted ‘Ref’) and the TIR and BRW modes (denoted ‘TE_0_’ and ‘BRW’, respectively) of the BRW device.

To tackle the problem of excessive losses, we use a transverse Bragg reflector approach^26^ previously explored in the context of traditional laser diodes^27,28^. In this approach, an optical gain medium is flanked with a DBR stack on one or both sides^26^ (Fig. 2b, left). The resulting Bragg reflection waveguide (BRW) supports low-loss modes (Extended Data Figs. 3 and 4) that develop owing to coherent superposition of several reflections produced by the DBR structure (Fig. 2b, left). The BRW mode is favoured over the TIR modes in the case of ASE as they offer improved mode confinement within the gain-active medium and, as a result, feature reduced optical losses and enhanced net modal gain^27,28^. Furthermore, the BRW mode is characterized by an increased effective amplification length, as the corresponding angle of incidence (*θ*_BRW_) can be considerably sharper than that in the TIR case (Fig. 2a,b, left and Extended Data Figs. 3c and 4b).

**Fig. 3:**
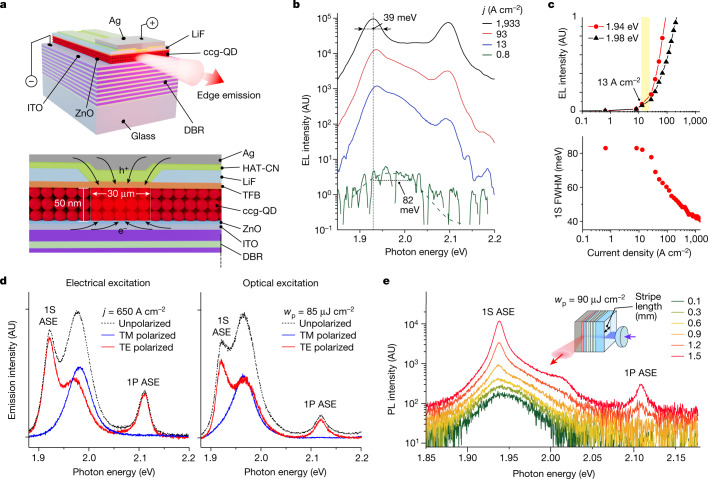
Electrically driven ASE in the BRW device. **a**, A BRW device is built on top of a DBR made of ten pairs of Nb_2_O_5_ and SiO_2_ layers. The device contains an ITO cathode, a ZnO ETL, a ccg-QD gain medium (three QD monolayers), a TFB HTL, a LiF interlayer with a current-focusing slit, a HAT-CN HIL and a strip-like Ag anode. **b**, Edge-emitted EL spectra of the BRW device as a function of current density tuned from 0.8 to 1,933 A cm^−2^. The device was excited using pulsed bias with *τ*_p_ = 1 μs and pulse-to-pulse separation *T* = 1 ms. The EL spectra show a transition from broad spontaneous emission observed at low *j* to sharp 1S and 1P ASE bands at high *j*. **c**, Top, the *j*-dependent EL intensities at the peaks of the 1S spontaneous (black) and ASE (red) bands indicate the ASE threshold *j*_th,ASE_ ≈ 13 A cm^−2^. Bottom, the dependence of 1S emission linewidth on *j* indicates progressive line narrowing from 82 to 39 meV. **d**, Polarization characteristics of edge-emitted light of the BRW device in the case of electrical (left, *j* = 650 A cm^−2^) and optical (right, 110-fs, 3.6-eV pulses, *w*_p_ = 85 μJ cm^−2^) excitation. Owing to strong damping of TM modes, the 1S and 1P ASE bands are not present in TM-polarized emission (blue) and exhibit nearly perfect TE polarization (red). The spontaneous 1S band is not polarized (black) and, as a result, is present in both TE-polarized and TM-polarized emission. **e**, The VSL measurements of the optically excited BRW device (inset) indicate the development of the 1S and 1P ASE features with increasing stripe length. These measurements used 110-fs, 3.6-eV pump pulses with *w*_p_ = 90 μJ cm^−2^. The sharp ASE bands are similar to those observed in the EL spectra (panel **b**). AU, arbitrary units.

To implement a BRW waveguide, we incorporate a DBR stack made of ten pairs of Nb_2_O_5_ and SiO_2_ layers below the cathode (Fig. 3a and Supplementary Fig. 4). To reduce serial resistance and thereby lessen overheating at high *j*, we make the cathode of standard ITO rather than higher-resistivity L-ITO used in refs. ^12,13^. As a result, we can push the current density up to 1,933 A cm^−2^ (*V* = 53 V) without causing device breakdown (Supplementary Fig. 5). To further improve charge flow in the device, we deposit an *n*-type ZnO electron-transport layer (ETL) on top of the ITO cathode (Fig. 3a). The ZnO ETL is followed by the QD layer and a series of hole transport/injection layers that are similar to those of the reference LED (Fig. 3a).

As well as improving charge transport, the ZnO layer also allows us to achieve *n*-doping of the active medium, as ZnO is known to facilitate electron injection into the QDs and thereby helps keep them negatively charged^29,30^. As shown previously, the use of charged (doped) QDs benefits lasing performance by lowering optical gain thresholds owing to partial or complete bleaching of ground-state absorption^31–35^. A potential problem of this approach is quenching of QD emission resulting from Auger recombination of charged excitonic species^32,33^. However, it is less of a problem with our ccg-QDs because, owing to impeded Auger decay, these QDs show high emission efficiencies for both singly and doubly negatively charged excitons (Supplementary Fig. 2).

In the fabricated structures, the bottom DBR and the top Ag mirror form a BRW. The computed electric-field distribution for the BRW mode is depicted in Fig. 2b, left. It exhibits an oscillatory pattern that reflects the periodic structure of the DBR. The main peak is centred within the QD optical gain medium, which leads to a high mode confinement factor (*Γ*_QD_ = 0.2), despite the small thickness of the gain medium (approximately three ccg-QD monolayers). Notably, the BRW mode profile also features a diminished field intensity in the optical lossy ITO and ZnO layers. As a result, the overall loss coefficient is only 16 cm^−1^ (Extended Data Fig. 4d).

The favourable changes in the optical-field distribution have a profound effect on device EL performance. In particular, we observe a marked boost in edge emission, whose intensity becomes greater than that of surface emission by a factor of around 2 to 3 (Fig. 2b, right). This is a direct consequence of the reduced propagation losses and the emergence of the regime of ASE. The effect of ASE is pronounced in the spectra of edge-emitted EL (Fig. 3b). At low injection levels (*j* < 8 A cm^−2^), they show a weak, single-band 1S emission at 1.98 eV with an 82-meV linewidth (full width at half maximum, FWHM). At higher current densities, we observe the emergence of new narrow features whose spectral energies (1.94 and 2.09 eV) are identical to those of the 1S and 1P ASE bands in the optically excited ccg-QD film (Fig. 1c). The new bands exhibit fast superlinear growth with increasing injection level (Supplementary Fig. 6) and eventually (at *j* ≥ 13 A cm^−2^) overtake the broad 1S band (Fig. 3c, top). This is accompanied by the pronounced narrowing of the band-edge emission from 82 to 39 meV (or 23 to 13 nm; Fig. 3c, bottom). The observed *j*-dependent evolution of the EL spectra is very different from that for the reference LED (Fig. 1f) but very similar to the evolution of photoluminescence (PL) during the transition to ASE for the optically excited ccg-QD/glass sample (Fig. 1c). This suggests that the narrow 1S and 1P features in the edge-emitted EL are also linked to ASE.

To infer the ASE threshold, we compare the *j*-dependent EL signals at 1.98 eV and 1.94 eV (Fig. 3c, top), which correspond to peak energies of the spontaneous emission and ASE, respectively. Although initially the two signals grow synchronously with increasing injection level (approximately linear), they start to diverge at *j* > 13 A cm^−2^ owing to the onset of faster (superlinear) increase of the 1.94-eV EL intensity (Supplementary Fig. 6). We ascribe this behaviour to the onset of ASE and the corresponding current density to the ASE threshold (*j*_th,ASE_ = 13 A cm^−2^). The value of *j*_th,ASE_, determined in this way, is consistent with the onset of line narrowing, characteristic of the ASE process (Fig. 3c, bottom).

The calculated ASE thresholds for our ccg-QD films depend on a charging level^33^ (Supplementary Note 2). For neutral QDs, *j*_th,ASE_ is about 28 A cm^−2^ and it drops to about 26 A cm^−2^ and then about 15 A cm^−2^ for singly and doubly negatively charged QDs, respectively. The comparison of these values with *j*_th,ASE_ observed experimentally suggests that, in our devices, QDs are populated with two electrons on average, which is consistent with previous studies of high-brightness cg-QD LEDs containing a ZnO ETL^29^.

**Extended Data Fig. 5:**
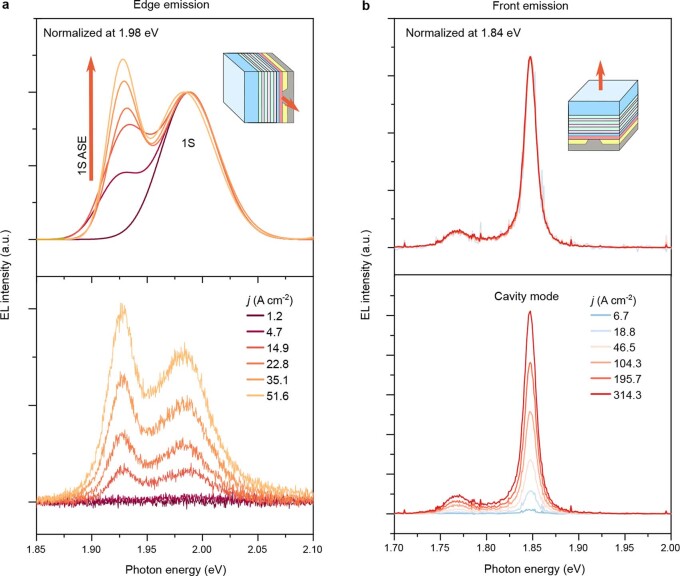
Evolution of edge-emitted and surface-emitted EL spectra with increasing current density. **a**, Edge-emitted EL spectra of the BRW device as a function of *j*. The bottom subpanel shows ‘raw’ (not normalized) experimental spectra. The top subpanel shows the normalized spectra scaled so as to match the amplitude of the 1S spontaneous emission feature (owing to a high noise level below the ASE threshold, we present the measured spectra using two-band Gaussian fits). The normalized spectra clearly show the emergence of a sharp 1S ASE band. **b**, Similar sets of data for surface-emitted EL of the same device. The EL is dominated by a vertical cavity mode at 1.84 eV. It is red-shifted versus that of the device shown in Extended Data Fig. 2b, because of a larger thickness of the device tested in the present measurements. The feature around 1.77 eV results from light leakage through the DBR (see Supplementary Fig. 4). Unlike edge-emitted EL, the surface-emitted signal shows spectrally uniform growth with increasing *j*. a.u., arbitrary units.

Next, we describe evidence that the sharp 1S and 1P EL features are indeed because of photon amplification during light propagation in the BRW and not because of spectral filtering effects arising, for example, from the DBR–Ag cavity. The first piece of evidence is the close correspondence between spectral positions of the EL peaks with the optically excited 1S and 1P ASE features observed for cavity-free ccg-QD/glass samples (Fig. 1c). Second, the comparison of surface-emitted and edge-emitted EL spectra (Extended Data Fig. 2b) shows that the ASE features are spectrally distinct from the vertical cavity mode. Furthermore, the edge-emitted and surface-emitted bands show distinct behaviours as a function of *j* (Extended Data Fig. 5). In particular, owing to the onset of ASE, edge-emitted EL shows spectrally non-uniform intensity growth, whereas such spectral non-uniformity is absent in the surface emission.

**Extended Data Fig. 6:**
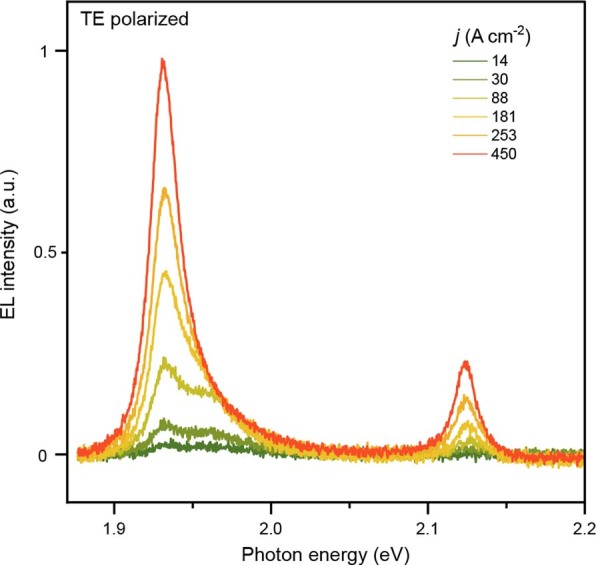
The *j*-dependent spectra of edge-emitted EL of  the BRW device measured with a TE polarizer. Owing to a ‘lossy’ character of TM modes (strongly attenuated by the silver electrode), ASE occurs because of low-loss TE modes. The spontaneous emission does not exhibit preferred polarization. Therefore, the use of a TE polarizer allows us to emphasize narrowband ASE features over broadband spontaneous emission. As a result, the EL spectra measured at high current densities (*j* > 200 A cm^−2^) are dominated by narrow 1S and 1P ASE peaks. The marked difference of these spectra from those of the reference devices (Fig. 1f and Extended Data Fig. 1a) is yet another confirmation of the ASE effect realized in our BRW devices. a.u., arbitrary units.

**Extended Data Fig. 7:**
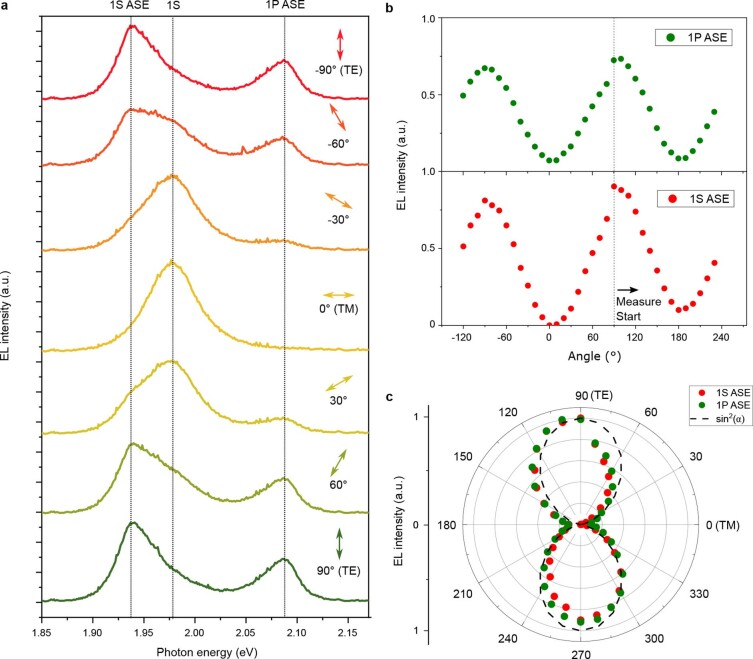
Polarization measurements of edge-emitted EL. **a**, Edge-emitted EL spectra of the BRW device operated at about 100 A cm^−2^ collected through a linear polarizer whose polarization direction is varied from 90° (vertical polarization; TE mode) to 0° (horizontal polarization; TM mode). The dashed black lines at 1.93, 1.98 and 2.08 eV correspond to the maxima of the 1S ASE, 1S spontaneous emission and 1P ASE features, respectively. Polarization-dependent changes in the EL spectral shape indicate that both the 1S and 1P ASE bands are strongly TE polarized. **b**, The amplitudes of the 1S ASE (red circles) and 1P ASE (green circles) bands as a function of polarization angle (*α*). The measurement of one data point took around 1 min and it took approximately 36 min to obtain the entire dataset. There was some degree of signal degradation during the measurements, which led, in particular, to a drift of a baseline. **c**, The same measurements plotted using polar coordinates. The experimental data (symbols; same colours as in **b**) are in good agreement with the theoretical sin^2^*α* dependence expected for TE-polarized light (dashed black line). a.u., arbitrary units.

Polarization-dependent measurements provide further evidence for the ASE regime. In particular, both sharp EL peaks observed at high *j* (post ASE threshold) are TE polarized and not present in TM-polarized emission (Fig. 3d and Extended Data Fig. 6). The detailed polarization-dependent measurements of the 1S and 1P EL features, ascribed to ASE, show a nearly perfect sin^2^*α* pattern, as expected for TE-polarized light (Extended Data Fig. 7; *α* is the angle between the polarization direction of the analyser and the vertical direction)^18^. This type of polarization is expected for amplified guided BRW modes, as propagation of TM modes is strongly inhibited owing to quenching by the Ag electrode^12,13^. Notably, the observed polarization trends are identical between the regimes of electrical and optical pumping (Fig. 3d; left and right subpanels, respectively). This is strong evidence for the ASE character of edge-emitted EL, as the ASE effect is unambiguous in optically excited edge-emitted PL spectra, as discussed below.

In Fig. 3e, we show VSL measurements of BRW structures conducted with optical excitation (see Methods). For these measurements, we prepare devices without a LiF spacer, which allows us to avoid parasitic signals from the parts of the QD layer outside the current-focusing aperture. In the VSL experiment, the pump laser beam is focused into a narrow stripe of a varied length (*l*), which is orthogonal to the cleaved device edge. For short stripe lengths, the edge-emitted PL is characterized by a broad spectral profile that is similar to that of EL at low injection levels (Fig. 3b,e; green lines). As *l* is increased, the emission intensity experiences quick growth (Supplementary Fig. 7), which is accompanied by the development of sharp peaks (Fig. 3e) whose spectral energies are in close agreement with the narrow EL features emerging at high *j* in electrically pumped devices (Fig. 3b, solid lines), as well as the 1S and 1P ASE bands observed for the optically excited ccg-QD/glass sample (Fig. 1c). First, these results exclude that the narrow 1S and 1P features arise from spontaneous emission of higher-order multiexcitons, as the increase in *l* does not affect per-pulse fluence, the quantity that controls the excitonic occupancy of the QDs. Second, these observations confirm the connection of the sharp 1S and 1P peaks to the process of stimulated emission, as the build-up of ASE does require a sufficiently long light propagation path in the gain medium approximately defined by the condition *G*_net_*l* > 1.

On the basis of the analysis of the *l*-dependent emission intensities, the 1S and 1P gain coefficients are 45 and 55 cm^−1^, respectively (Supplementary Fig. 7). These values are close to the calculated maximal net optical gain for charged QDs (*G*_net_ = 0.5*G*_mod,max_ − *α*_loss_ ≈ 64 cm^−1^; Supplementary Note 2), in agreement with our earlier analysis of ASE thresholds, according to which the observed gain is because of charged excitons.

**Extended Data Fig. 8:**
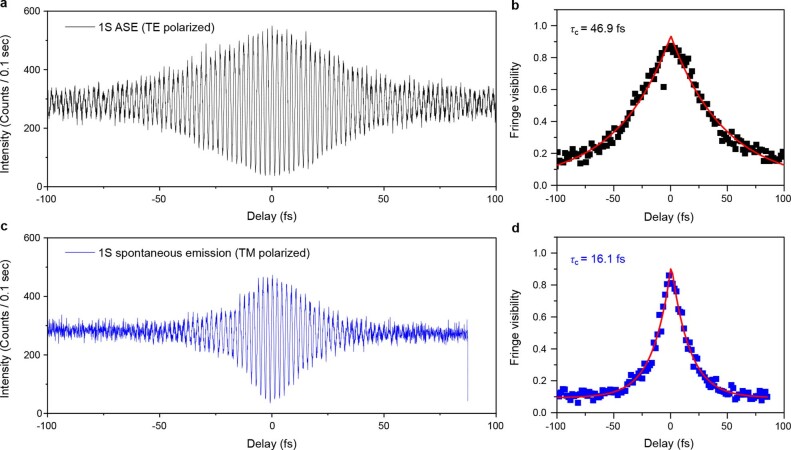
Coherence measurement of edge-emitted EL conducted using Michelson interferometry. **a**, Interferogram of the 1S ASE band of the BRW device driven at about 100 A cm^−2^. The edge-emitted light is fibre coupled into a Michelson interferometer consisting of a cube beam splitter, a fixed arm and a variable-length arm controlled by a piezoelectric stage. The interference signal is detected with a Si avalanche photodiode. 1S ASE is isolated using a 620-nm (or 2-eV) long-pass filter and a linear polarizer set to the ‘vertical’ (TE) polarization (Fig. 3d, left). **b**, Fringe visibility extracted from the interferogram in **a** (black symbols) as a function of the time delay between the two arms of the interferometer. An exponential fit of the experimental data (red line) yields coherence time *τ*_c_ of 47 fs. **c**,**d** The interferometric measurements conducted for the ‘horizontal’ (TM) polarization in which ASE is suppressed (Fig. 3d, left) yield a much shorter coherence time of 16 fs. The observed polarization-dependent threefold difference in *τ*_c_ indicates the presence of two subsets of photons with distinct temporal coherences. As expected for the ASE effect, the photons produced by stimulated emission (they dominate the edge-emitted TE-polarized light) exhibit a longer *τ*_c_ compared with photons produced by spontaneous emission (they dominate the edge-emitted TM-polarized light). The measured coherence times are consistent with those inferred from *τ*_c_ ≈ (πΔ*ν*)^−1^, in which Δ*ν* is the emission linewidth in the frequency domain. In particular, based on the linewidths of the 1S spontaneous and ASE bands (82 to 30 meV, respectively, for the sample used in the studies of coherence), the estimated coherence times are 16 fs and 44 fs. Both values are in excellent agreement with the results of the interferometric measurements (16 fs and 47 fs, respectively).

The effect of ASE is also evident in the measurements of temporal coherence conducted using a Michelson interferometer. In particular, under conditions similar to those in Fig. 3d, left, the coherence time (*τ*_c_) observed for the TE-polarized light is appreciably longer (by a factor of about three) than that for the TM-polarized EL (Extended Data Fig. 8). The lengthening of *τ*_c_ indicates a considerable contribution of ASE to the TE-polarized EL, as photon replication during light amplification enhances temporal coherence. These results are consistent with the measurements of spectrally resolved EL that indicate the dominance of ASE in the TE-polarized emission (Fig. 3d, left and Extended Data Fig. 6).

**Fig. 4:**
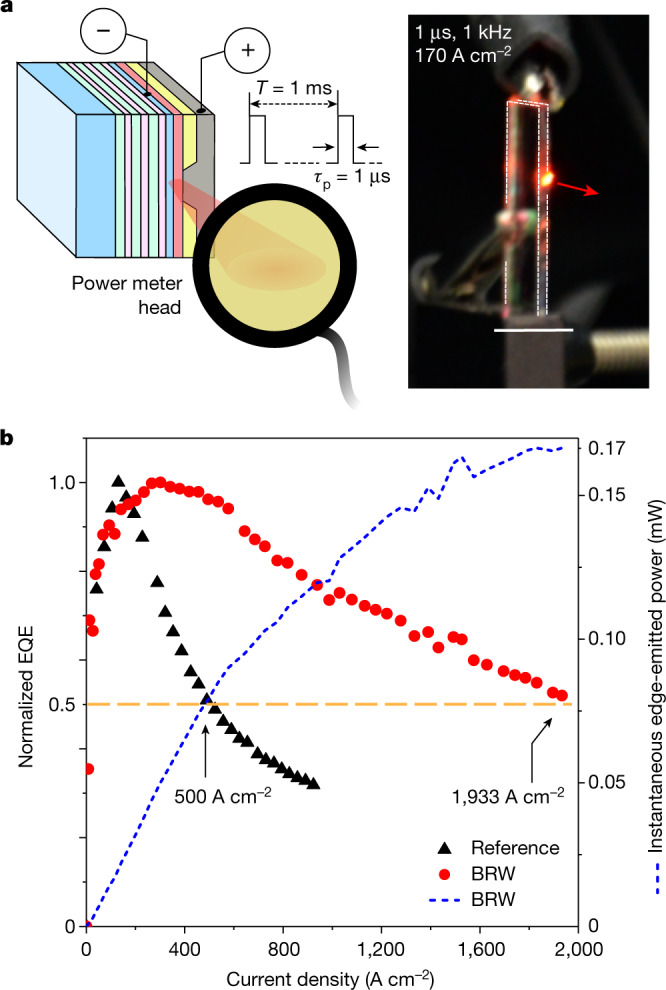
Characterization of the output of the BRW device. **a**, Left, the output of the electrically excited BRW device was characterized using a standard laser-lab power meter. The measured average power (*P*_av_) was converted into the instantaneous output power (*P*_out_) using *P*_out_ = (*T*/*τ*_p_)*P*_av_. For *τ*_p_ = 1 μs and *T* = 1 ms used in our experiments, *P*_out_ = 1,000*P*_av_. Right, photograph of the device operating at *j* = 170 A cm^−2^ under ambient conditions in room light clearly shows edge emission that appears very bright despite the small on-time fraction (*τ*_p_/*T* = 0.001) and the small size of the emitting spot (the nominal area is about 9 μm^2^). Scale bar, 10 mm. **b**, The dashed blue line shows the *j*-dependent instantaneous output power. At the maximal current density (*j* = 1,933 A cm^−2^), *P*_out_ reaches 170 μW. On the basis of the measured output power, we determine the EQE (red circles), which is compared with that of the reference device (black triangles). Owing to the efficient ASE, which leads to the increased QD emission rate and enhanced power extraction from the inverted QD medium, the EQE droop is much less pronounced in the BRW device. In particular, *j*_½_ is about four times higher than that for the reference device (1,933 versus 500 A cm^−2^).

As pointed out earlier, another indication of the ASE in the BRW structures is the high brightness of edge-emitted EL (Fig. 2b, right). In the reference device, the edge signal is undetectable by the naked eye, even in the dark. By contrast, as illustrated in Fig. 4a, the light radiating from the edge of the BRW device is clearly seen even in room light, despite a very small edge-emitting area (its nominal size is approximately 9 μm^2^). In fact, the emission from the BRW structure can be detected and characterized with a standard power meter used to evaluate the output of commercial lasers. On the basis of such characterization, the instantaneous edge-emitted power (*P*_out_) during the voltage pulse reaches 170 μW (*j* = 1,933 A cm^−2^); Fig. 4b (dashed blue line). A substantial role in the development of strong edge-emitted ASE is played by the BRW structure, which increases the effective amplification length and improves the collection of ‘seed’ photons produced by spontaneous emission (Supplementary Fig. 8).

The edge-emitted light exhibits a fairly tight angular distribution for out-of-plane angles (Supplementary Fig. 9a,b). It features a sharp spike (from approximately −0.2° to 0.2°), which appears on top of an asymmetric profile extending to the DBR device side. Such asymmetry is consistent with the calculated BRW mode structure (Fig. 2b, left). The angular distribution for in-plane angles is fairly flat (Supplementary Fig. 9c,d), as expected for our devices that lack angle-selection elements in the device plane.

**Extended Data Fig. 9:**
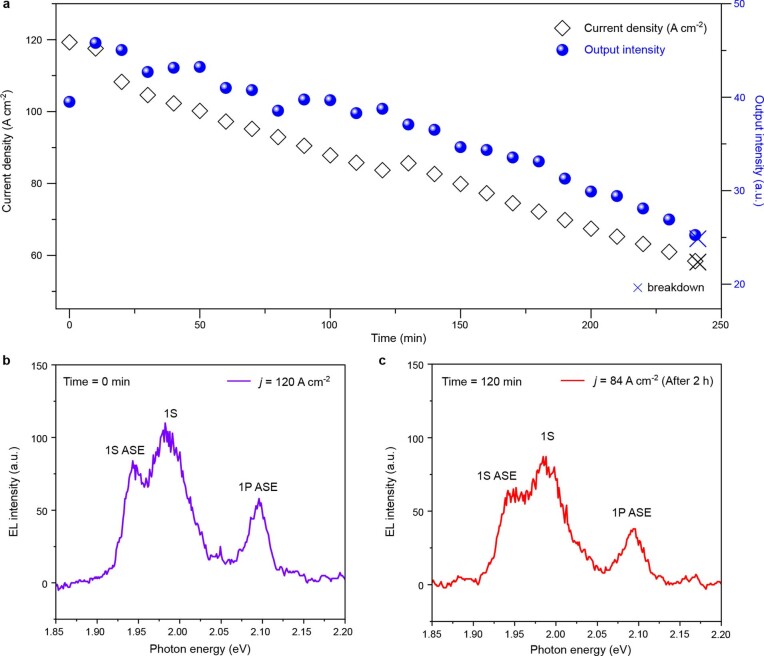
Studies of operational stability of a BRW device. **a**, The dependence of the current density (left axis, open diamonds) and the output power (right axis, blue circles) of an unencapsulated BRW device on time of operation under excitation with 10-V, 1-µs pulses at 1 kHz. The data were recorded at intervals of 1 min. The data points shown in the plot (separated by 10-min intervals) are averages over ten measurements. During the first 2 h, the output intensity decreased by only 9%. A slow decrease in the output power and the current density continued for the next 2 h, at which point device failure finally occurred. On the basis of these measurements, the characteristic *T*_75_ time was 2.7 h (*T*_75_ denotes the time at which the device output decreases to 75% of its original value). **b**, The EL spectra at the beginning of the test show distinct 1S and 1P ASE features. **c**, These features are preserved following 2 h of continuous device operation. a.u., arbitrary units.

The fabricated devices exhibit good operational stability under ambient environment. Even when the driving voltage is well above the ASE threshold, they operate for hours in the ASE regime without considerable losses in the output power. In particular, a stability test conducted at *j* = 120 A cm^−2^ (at the beginning of the test) shows that, after 2 h of continuous operation, the device still preserves around 90% of its original power (Extended Data Fig. 9). It operates in the stable ASE mode for two more hours, at which point the device finally fails.

**Extended Data Fig. 10:**
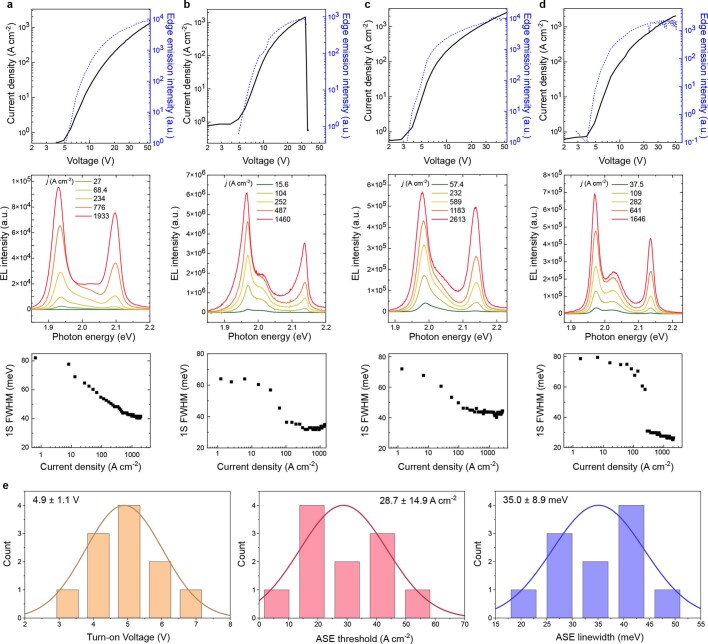
Reproducibility of characteristics of BRW ASE devices. **a**–**d**, Characteristics of four devices from four different chips (each chip contains eight devices). The data are organized into four columns, one column per device. The top row shows the *j*–*V* curves (black lines) and the *V*-dependent edge emission intensity (right axis, blue lines). The middle row shows EL spectra that feature prominent 1S and 1P ASE bands. The bottom row shows the dependence of the 1S bandwidth on *j*, which exhibits ‘line narrowing’ typical of the transition to the ASE regime. There is excellent consistency between all shown datasets. **e**, The analysis of device-to-device variability of a turn-on voltage (left), a 1S ASE threshold (middle) and a 1S ASE linewidth. The histograms were obtained on the basis of the measurements of 11 devices. The average values of the measured parameters and the standard deviations are indicated in the figure. In the case of the turn-on voltage and the ASE linewidth, the standard deviations are 22% and 25% of the average value, respectively. The larger deviation observed for the ASE threshold (about 52%) can be attributed to high sensitivity of *j*_th,ASE_ to device-to-device variations in propagation losses and a varied degree of charging of an active QD layer. a.u., arbitrary units.

Overall, we have fabricated 15 chips, each of which contained eight devices (120 devices in total). We observed excellent reproducibility of performance characteristics between devices on the same chip and those on different chips prepared through separate fabrication cycles. In particular, high-*j* EL measurements were conducted on 11 devices from different chips. All of them showed the ASE effect. As illustrated in Extended Data Fig. 10, the tested devices exhibited good consistency between their *j*–*V* (*P*_out_–*V*) dependences, EL spectra, ASE thresholds and the characteristic line narrowing accompanying the transition to the ASE regime.

It is instructive to examine the external quantum efficiency (EQE) of the BRW device versus the reference LED. Because our devices lack lateral optical confinement within the QD layer and do not use any schemes for improved light outcoupling, the collected edge-emitted light is only a small fraction of the total ASE. Therefore, we will focus on the analysis of normalized EQEs as a function of current density (Fig. 4b). For the reference device, the EQE reaches its peak value at *j* of about 130 A cm^−2^, after which it shows a fast decline and drops to half of the maximal value at *j* = *j*_½_ = 500 A cm^−2^ (Fig. 4b, black triangles). This is the manifestation of a droop effect typically attributed to processes such as nonradiative Auger recombination and/or thermally induced emission quenching^10,29^. The BRW device also exhibits the EQE droop. However, its onset is shifted to about 300 A cm^−2^ and *j*_½_ is increased to about 1,930 A cm^−2^ (Fig. 4b, red circles). These are expected consequences of the ASE regime, which accelerates radiative recombination and thus allows it to compete more favourably with nonradiative processes.

In conclusion, we demonstrate 1S and 1P ASE with an electrically excited gain medium made of solution-cast colloidal QDs. This advance has been enabled by excellent optical-gain properties of ccg-QDs and a specially engineered device stack, which contains a low-loss photonic waveguide. This waveguide is formed by the bottom DBR and the top Ag mirror that flank the QD medium and the adjacent charge transport/injection layers. The use of the BRW allows us to shape the optical-field profile so as to reduce optical losses in charge-conducting layers and enhance mode confinement in the QD medium. These ASE diodes exhibit strong edge emission with instantaneous output power of up to 170 μW, even though they lack lateral optical confinement within the gain-active region and do not use engineered light outcoupling. The next important milestone—the realization of a QD laser oscillator—can be accomplished by supplementing the developed structures with an optical resonator implemented, for example, as either an in-plane distributed feedback grating or a Fabry–Pérot cavity formed by edge reflectors.

## Methods

### Materials

Cadmium acetate dihydrate (Cd(CH_3_COO)_2_·2H_2_O, 98%), zinc acetate dihydrate (Zn(CH_3_COO)_2_·2H_2_O, 99.99%), selenium (shot, 2–6 mm, 99.998%) and sulfur (99.999%) were purchased from Alfa Aesar. Oleic acid (OA, 90%), 1-octadecene (ODE, 90%), toluene (anhydrous, 99.8%), chloroform (anhydrous, 99%), 2-propanol (anhydrous, 99.5%), ethanolamine (99.5%) and octane (anhydrous, 99%) were purchased from Sigma-Aldrich. Trioctylphosphine (TOP, 97%) was purchased from Strem. Ethanol (absolute, 200 Proof) was purchased from Fisher Chemical. TFB and HAT-CN were purchased from Lumtec. Silver pellets (Ag, 99.99%) were purchased from Kurt J. Lesker. The materials were used as received.

### Synthesis of ccg-QDs

#### Preparation of precursor solutions

The synthesis of CdSe/Cd_*x*_Zn_1−*x*_Se/ZnSe_0.5_S_0.5_/ZnS ccg-QDs was conducted using four precursor solutions (A, B, C and D). Solution A was prepared by mixing 0.5 ml Cd-oleate (0.5 M), 0.125 ml TOP-Se (2 M) and 0.375 ml ODE. The 0.5 M Cd-oleate solution was prepared in a three-neck flask by dissolving 10 mmol Cd(CH_3_COO)_2_·2H_2_O in 10 ml OA and 10 ml ODE. The cadmium mixture was degassed at 120 °C under vacuum for 1 h and kept at 100 °C under nitrogen for further use. The 2 M TOP-Se solution was prepared in a glove box by dissolving 40 mmol selenium in 20 ml TOP. Solution B was prepared by mixing 1.25 ml Cd-oleate (0.5 M), 1.25 ml TOP-Se (2 M) and 2.5 ml ODE. Solution C was prepared by mixing 1 ml ODE, 0.5 ml TOP-Se (2 M) and 0.5 ml TOP-S (2 M). The 2 M TOP-S was prepared in a glove box by dissolving 40 mmol sulfur in 20 ml TOP. Solution D was prepared by mixing 0.5 ml ODE and 0.5 ml TOP-S (2 M).

#### Synthesis

The synthesis of the ccg-QDs started with the preparation of CdSe cores. For this purpose, 6 ml ODE and 0.2 ml Cd-oleate (0.5 M) were loaded into a 100-ml three-neck flask and degassed at 120 °C for 25 min. The reaction flask was heated to 310 °C under nitrogen and 0.1 ml TOP-Se (2 M) was promptly injected into the reaction. Forty seconds after TOP-Se injection, 1 ml TOP was added dropwise for 20 s. In 2 min, 1 ml of solution A was added continuously to the reaction flask at a rate of 5 ml h^−1^ for 12 min.

In the next step, the preformed core particles were overcoated with a compositionally graded Cd_*x*_Zn_1−*x*_Se layer. For this purpose, 2 ml Zn-oleate (0.5 M) solution was promptly injected into the reaction flask at a temperature of 310 °C. 0.5 M Zn-oleate solution was prepared beforehand by mixing 20 mmol Zn(CH_3_COO)_2_·2H_2_O, 20 ml OA and 20 ml ODE in a three-neck flask. The mixture was degassed at 130 °C for 1 h under vacuum and then kept at 120 °C. After the injection of Zn-oleate, 5 ml of solution B were continuously added into the reaction flask at a rate of 4 ml h^−1^ for 75 min. While adding solution B, Zn-oleate (0.5 M) solution was promptly injected three times at 18.75, 52.5 and 67.5 min in the amounts of 2, 4 and 2 ml, respectively. Next, the ZnSe_0.5_S_0.5_ shell was grown on top of the Cd_*x*_Zn_1−*x*_Se layer. During this reaction stage, 1.5 ml of solution C was continuously added into the reaction flask at 310 °C at a rate of 2 ml h^−1^ for 45 min. While adding solution C, Zn-oleate (0.5 M) solution was promptly injected three times at 15, 30 and 45 min in the amount of 1 ml per injection. The particles were completed with a thin ZnS layer. 0.5 ml of solution D was continuously added in the reaction flask at 310 °C at a rate of 1 ml h^−1^ for 30 min. While adding solution D, 1 ml Zn-oleate (0.5 M) solution was promptly injected once at 15 min. The reaction flask was cooled to room temperature by removing the heating mantle and adding 36 ml chloroform at 70 °C. This synthesis resulted in the ccg-QDs schematically depicted in Fig. 1a.

#### Purification

The synthesized ccg-QDs were purified with ethanol by centrifuging at 7,000 rpm for 5 min and then redispersing in 10 ml toluene. These solutions were used in spectroscopic measurements. For device fabrication, ccg-QDs were further washed with acetonitrile. In this procedure, 2 ml of ccg-QDs in toluene were mixed with 20 ml acetonitrile and centrifuged at 9,000 rpm for 15 min. The washing step was repeated two more times. The precipitate was fully dried and redispersed in octane to obtain a desired concentration (typically, 20 mg ml^−1^).

### Fabrication of reference LEDs

Glass substrates coated with L-ITO were purchased from Thin Film Devices, Inc. The glass/L-ITO substrate was washed using sequential 10-min sonication steps in isopropyl alcohol, acetone and ethanol. After the cleaning step, the substrate was dried using a N_2_ gas blower. Afterwards, 20 μl of ccg-QD solution (20 mg ml^−1^) were spin-coated onto the L-ITO substrate at 2,000 rpm for 30 s to form one monolayer of the ccg-QDs. This procedure was repeated two more times to prepare a film that nominally contained three ccg-QD monolayers. Following deposition, the ccg-QD film was annealed at 100 °C for 10 min. To fabricate a HTL, 10 mg of TFB were dissolved in 1 ml of chlorobenzene and spin-coated onto the ccg-QD layer at 4,000 rpm for 30 s, which was followed by annealing at 120 °C for 20 min. Then, a 50-nm-thick LiF interlayer was deposited by thermal evaporation using a shadow mask, which defined a ‘current-focusing’ aperture in the form of the 30-μm-wide slit. After that, a 100-nm-thick HIL of HAT-CN was deposited using thermal evaporation with a deposition rate of 0.2–0.3 Å s^−1^. The device was completed with a 100-nm-thick Ag electrode deposited by means of thermal evaporation (at a rate of 1 Å s^−1^) through a shadow mask with a 300-μm-wide slit orthogonal to the slit in the LiF interlayer. This allowed us to obtain two-dimensional current focusing and limit the injection area to 30 × 300 μm^2^. We would like to point out that the hole-injection part of our devices is distinct from that of traditional QD LEDs that usually use a combination of MoO_*x*_ HIL and an organic HTL. However, the standard HIL/HTL combination leads to large optical losses that are mitigated here using the new design of the hole-injection device part^12,13^.

### Fabrication of devices with a BRW

BRW devices were assembled on top of ITO-coated DBR substrates purchased from Thin Film Devices, Inc. The substrates were custom made to match their stopband to the emission spectra of the ccg-QDs. In particular, their reflection coefficient was >95% (normal incidence) across the wavelength window of 490–690 nm (Supplementary Fig. 4), which covered both the 1S and 1P emission bands (Fig. 1c). The DBR was made of ten pairs of Nb_2_O_5_ and SiO_2_ layers (60 nm and 100 nm thickness, respectively) prepared on a glass substrate. A 50-nm-thick ITO film was deposited on top of the Nb_2_O_5_ layer of the DBR. The resulting multilayered stack is depicted in Supplementary Fig. 4. The acquired ITO/DBR/glass substrates were cleaned using the same procedure as in the case of reference devices. Then, a ZnO ETL with a thickness of 50 nm was deposited through a sol–gel method. A sol–gel solution was prepared by dissolving 0.2 g of zinc acetate dihydrate (Zn(CH_3_COO)_2_·2H_2_O) and 56 mg of ethanolamine in 10 ml of 2-methoxyethanol (CH_3_OCH_3_CH_3_OH). The solution was stirred overnight before use. 300 μl of a sol–gel precursor was spun at 3,000 rpm for 50 s and annealed at 200 °C for 2 h in ambient air. Afterwards, the active ccg-QD layer and the rest of the device were prepared using the same steps as in the case of reference LEDs (see previous section).

### Device characterization

All fabricated devices were tested at room temperature in air. For edge-emission measurements, devices were cleaved across the emitting area using a diamond tip. In the regime of electrical excitation, the devices were driven using square-shaped voltage pulses generated by a function generator (Tektronix AFG320; pulse amplitude up to 3.5 V), followed by a high-speed bipolar amplifier (HSA4101, NF Corporation) with 20 times voltage gain. The voltage applied to a device was measured using a Tektronix oscilloscope (TDS2024B) connected to the monitoring port of the amplifier. The generated transient current was measured by monitoring the voltage drop across a 10-Ω-load resistor on the current return (Supplementary Fig. 5).

Both edge-emission and front-emission spectra were collected using a Czerny–Turner spectrograph (Acton SpectraPro 300i) dispersing light in the focal plane of a liquid-nitrogen-cooled charge-coupled device (CCD) camera (Roper Scientific) or a fibre-coupled Ocean Optics USB 2000 spectrometer (Fig. 1e,f, Fig. 3b and Extended Data Fig. 10). The spectral resolutions were 0.1 nm and 0.4 nm, respectively. The optical power of edge emission was measured using a standard photodiode-based power meter (Thorlabs S120VC with an active area of 73 mm^2^). The power meter head was positioned 1 cm away from the cleaved edge of the device (Fig. 4a). The EQE was obtained on the basis of the instantaneous output power emitted during the voltage pulse (*P*_out_) and the driving current (*I*) using the following expression:

$${\rm{EQE}}=\frac{({P}_{{\rm{out}}}/hv)}{(I/{\rm{e}})}$$

in which *hv* is the averaged energy of the edge-emitted photons calculated from the measured EL spectra and e is the elementary charge.

### Optical measurements

#### Optical absorption and PL measurements

Optical absorption and PL measurements were conducted on ccg-QD/toluene solutions loaded into 1-mm-thick quartz cuvettes. The absorption spectra were collected with an ultraviolet–visible scanning spectrometer (Lambda 950, Perkin Elmer). In the PL lifetime studies, a ccg-QD sample was excited with 3.1-eV, 40-fs pulses at a 250-kHz repetition rate derived from a frequency-doubled Ti:sapphire laser (Mira oscillator and RegA amplifier, Coherent). The laser pulses were focused onto the sample into a 100-µm-diameter spot. The emitted PL was collected in the direction normal to the sample plane, spectrally selected with a Czerny–Turner spectrograph (Acton SpectraPro 300i) equipped with an exit slit and detected with a fibre-coupled superconducting nanowire single-photon detector (Opus One, Quantum Opus), followed by a time-correlated single-photon counting apparatus (PicoQuant PicoHarp). The PL was measured at the maximum of the 1S PL peak with a 2-nm bandwidth; the temporal resolution of the setup was 70 ps.

#### TA spectroscopy

For transient absorption (TA) studies, ccg-QDs were diluted in toluene and loaded into a 1-mm-thick quartz cuvette to achieve an optical density of around 0.2 at 2.4 eV. The sample was continuously stirred during the measurements to avoid uncontrolled photocharging. A home-built pump–probe TA setup used a regeneratively amplified femtosecond ytterbium-doped potassium gadolinium tungstate (Yb:KGW) laser (PHAROS, Light Conversion) generating 190-fs, 1,030-nm  (1.2-eV) pulses. The repetition rate was set to 500 Hz. The laser output was split into a pump and a probe channel. The signal in the pump arm was frequency doubled in a BBO crystal to produce 110-fs, 515-nm (2.4-eV) pulses. The pump beam was focused onto the sample into a 130-µm spot. The probe pulses transmitted through a delay line were focused onto a sapphire plate to generate a white-light supercontinuum, which was then focused onto the sample into an 80-µm spot overlapping the centre of the sample area excited by the pump pulses. A mechanical chopper in the pump arm blocked every second pulse from a pump–pulse sequence. The transmitted probe pulses were analysed with an Avantes AvaSpec-ULS1350F-USB2 spectrometer synchronized with the chopper. The pump-induced changes in the absorption coefficient of a ccg-QD sample (Δ*α* = *α* − *α*_0_, in which *α* and *α*_0_ are the excited-state and ground-state absorption coefficients, respectively) were acquired as a function of pump fluences (tuned from 3 µJ cm^−2^ to 6 mJ cm^−2^) and pump–probe delay (varied from −5 ps to 4.5 ns).

#### TA data analysis and quantification of optical gain

The ‘chirp’ of the white-light supercontinuum probe was analysed and accounted for using procedures of ref. ^36^. The pump level was evaluated in terms of a per-pulse fluence (*w*_p_). The average per-dot excitonic occupancy generated by the pump pulse was inferred from ⟨*N*⟩ = *σ*_p_*w*_p_/*hv*_p_, in which *hv*_p_ is the pump-photon energy and *σ*_p_ is the corresponding ccg-QD absorption cross-section. The value of *σ*_p_ was determined from a Poisson analysis of *w*_p_-dependent long-delay TA signals following the procedure of ref. ^37^. Two-dimensional optical gain ‘maps’ in the photon energy–delay time (*hv*–*t*) space were obtained by computing excited-state absorption from *α*(*hv*,*t*) = *α*_0_(*hv*) + Δ*α*(*hv*,*t*). In this representation, optical gain appears as features with *α*(*hv*,*t*) < 0.

In this work, we use several metrics to evaluate optical gain that include material gain, modal gain and net gain. We define material gain (*G*_mat_) as a gain coefficient of an infinitely thick close-packed QD film. The corresponding mode confinement factor of the QD medium (*Γ*_QD_) is 1. Modal gain (*G*_mod_) is the gain coefficient of a QD film of a final thickness. It relates to *G*_mat_ by *G*_mod_ = *Γ*_QD_*G*_mat_, in which *Γ*_QD_ is the mode confinement factor of the QD film, which is less than 1. Net gain (*G*_net_) corresponds to the overall gain coefficient of a device that accounts for optical losses: *G*_net_ *=* *G*_mod_ − *α*_loss_, in which *α*_loss_ is the optical-loss coefficient.

#### ASE measurements

A ccg-QD film or a cleaved electroluminescent device was mounted in the focal plane of a cylindrical lens with a focal length of 10 cm. Femtosecond pulses (110-fs duration, 3.6-eV photon energy, 100-Hz repetition rate) from a tripled Yb:KGW laser (PHAROS, Light Conversion) were focused onto the sample into a 1.7-mm-long stripe orthogonal to the sample edge. The stripe width was approximately 40 μm. The edge-emitted light was collected with an imaging system and analysed using a Czerny–Turner spectrograph (Acton SpectraPro 300i) coupled to a liquid-nitrogen-cooled CCD camera (Roper Scientific). In VSL measurements, the length of the excited strip (*l*) was varied by moving a razor blade placed into the pump beam (Fig. 3e and Supplementary Fig. 3). To determine net optical gain (*G*_net_), the intensity of edge-emitted light (*I*_edge_) was fit to *I*_edge_ = *B*(exp(*G*_net_*l*) − 1)/*G*_net_ + *Cl*, in which *B* and *C* were *l*-independent constants.

#### Michelson interferometry measurements

To characterize the temporal coherence of a device output, edge-emitted light was collected and collimated with an objective (Olympus PLN 10X) and sent into a Michelson interferometer. A linear polarizer was used to select either ASE or spontaneous emission. The beam was split between two arms using a non-polarizing 50/50 beam splitter and reflected using flat mirrors. One mirror was mounted onto a single-axis linear stage (Aerotech ANT130L), which enabled accurate control of the time delay between the two arms. The output beams were collected using an objective (Olympus PLN 10X) and detected with an avalanche photodiode (Micro Photon Devices PDM series). A time-tagged, time-resolved mode provided by a time-correlated single-photon counting module (PicoQuant HydraHarp 400) was used to record the intensity of the interference pattern.

### Reporting summary

Further information on research design is available in the Nature Portfolio Reporting Summary linked to this article.

## Online content

Any methods, additional references, Nature Portfolio reporting summaries, source data, extended data, supplementary information, acknowledgements, peer review information; details of author contributions and competing interests; and statements of data and code availability are available at 10.1038/s41586-023-05855-6.

## Supplementary information

Supplementary Information This file contains two Supplementary Notes, Supplementary Figures 1–9 and extra references that extend and support the data and discussion presented in the main text.

Reporting Summary

## Data Availability

The data that support the findings of this study are available from the authors on reasonable request.
