# Supplementary material for: Electrically driven amplified spontaneous emission from colloidal quantum dots
Source: Nature. 2023 May 3;617(7959):79–85. doi: 10.1038/s41586-023-05855-6 (PMC10156592; doi:10.1038/s41586-023-05855-6)
Supplement: Supplementary file 1 — This file contains two Supplementary Notes, Supplementary Figures 1–9 and extra references that extend and support the data and discussion presented in the main text. [file 41586_2023_5855_MOESM1_ESM.pdf]

nature portfolio

https://doi.org/10.1038/s41586-023-05855-6

Supplementary information

Electrically driven amplified spontaneous emission from colloidal quantum dots

In the format provided by the authors and unedited

Supplementary Information for:

Electrically Driven Amplified Spontaneous Emission from Colloidal Quantum Dots

Namyoung Ahn[1], Clément Livache[1], Valerio Pinchetti[1], Heeyoung Jung[1,2], Ho Jin[1,2], Donghyo Hahm[1], Young-Shin Park[1], and Victor I. Klimov[1]*

[1]Nanotechnology and Advanced Spectroscopy Team, C-PCS, Chemistry Division, Los Alamos National Laboratory, Los Alamos, New Mexico 87545, USA

[2]Center for High Technology Materials, University of New Mexico, Albuquerque, NM 87106, USA

*klimov@lanl.gov

Supplementary Notes

Supplementary Note 1. Photonic calculations

Methodology. To model optical modes of waveguide structures, we applied a finite element method (FEM) implemented with the wave-optics module of COMSOL Multiphysics Software. The waveguide parameters were defined by those of multilayered device stacks studied experimentally. We did not take into account scattering losses due to waveguide imperfections.

In the description of the devices, we use the following abbreviations: quantum dot (QD), indium tin oxide (ITO), poly[(9,9-dioctylfluorenyl-2,7-diyl)-co-(4,4'-(N-(4-sec-butylphenyl) diphenylamine)) (TFB), and dipyrazino[2,3-f :2',3'-h]quinoxaline-2,3,6,7,10,11-hexacarbonitrile (HAT-CN).

The 'reference' device was defined as: glass (1 μm)/L-ITO (150 nm)/QDs (50 nm)/TFB (50 nm)/HAT-CN (100 nm)/Ag (100 nm); Figs. 1d and 2a of the main article.

The device based on a Bragg reflector waveguide (BRW) was defined as: glass (1 μm)/SiO_2 (100 nm):Nb_2O_5 (60 nm) (10 pairs)/ITO (50 nm)/ZnO (50 nm)/QDs (50 nm) /TFB (50 nm)/HAT-CN (100 nm)/Ag (100 nm); Figs. 2b and 3a of the main article.

The refractive indices (n) and extinction coefficients (k) of device layers were inferred from either the COMSOL material library or from published literature.[1,2] The QD layer was assumed to be optically transparent (k = 0) which corresponded to the situation of the optical-gain threshold.

Electric-field distribution. Here, we consider only transverse electric (TE) modes as transverse magnetic (TM) modes are strongly quenched by the silver electrode.[1] Computations were performed by solving the following frequency-domain wave equation:

$$\nabla^2 E - k_0^2 \mu_r \varepsilon_r E = 0$$

where $\mu_r = 1$ (no magnetic materials) and ε_r (relative permittivity) was computed from $\varepsilon_r = (n - jk)^2$. To solve this equation, we used 'perfect' electric-conductor boundary condition at the interface with the top Ag electrode, that is, we assumed that 100% of incident light was reflected at the Ag boundary. For the bottom glass layer, we applied the 'scattering-type' boundary conditions. In particular, we assumed that waves which were not reflected at the boundary (that is, fall into the 'escape' cone) were absorbed by it. To simulate waveguide modes, the left-side boundary of the waveguide structure was set as a wave input using 'numeric-port' boundary

conditions. The resulting solutions yield a spatial distribution of the electric field for waves propagating from the left to the right edge of the waveguide structure (Extended Data Fig. 3a).

Mode dispersion. To obtain dispersion of waveguide modes presented in Extended Data Fig. 3b, we used COMSOL software for an eigenmode analysis. The eigenmode solver provides eigenmodes of the wave equation based on a specific waveguide geometry and boundary conditions. The computed eigenmodes are characterized by an effective mode index, $n_{eff}(\omega)$, and a wavevector, $\beta = n_{eff}\omega/c$, where ω is the angular frequency and c is the speed of light in vacuum. We defined n_1 and n_2 as the lowest and the highest optical indices in the structure (in our case, these were the indices of SiO_2 and Nb_2O_5, respectively). To compute BRW modes, the solver was instructed to find possible modes in the photonic bandgap region, which corresponded to $n_{eff} < n_1$. Total internal reflection (TIR) modes were obtained using condition $n_1 < n_{eff} < n_2$.

Mode confinement factor and optical loss coefficient. Eigenvalues computed with the eigenmode solver were used in a frequency-domain solver to find the distribution of a three-component electric-field vector ($\boldsymbol{E}$) in the two-dimensional geometric domain. Then, the mode confinement factor of the QD layer (Γ_{QD}) was calculated from

$$\Gamma_{\mathrm{QD}} = \frac{\int_{\mathrm{QD}} |\boldsymbol{E}|^2 \mathrm{d}y\mathrm{d}z}{\int |\boldsymbol{E}|^2 \mathrm{d}y\mathrm{d}z},$$

where the integration extended over the QD layer (numerator) and the entire space (denominator). The waveguide loss coefficient (α_{loss}) was calculated based on the mode effective refractive index (n_{eff}) from

$$\alpha_{\mathrm{loss}} = -\frac{2\omega}{c} \times \mathrm{Im}(n_{\mathrm{eff}}).$$

Supplementary Note 2. Modeling of amplified spontaneous emission thresholds

Threshold modeling. To determine the threshold of amplified spontaneous emission (ASE), we use the following condition:

$$G_{mod} = \alpha_{loss}, \quad (1)$$

where G_{mod} is the modal gain coefficient and α_{loss} is the overall optical-loss coefficient. Below, we consider ASE thresholds for the situations of neutral, singly negatively charged, and doubly negatively charged QDs.

Neutral QDs. In the case of neutral QDs, the maximal (saturated) modal gain ($G_{mod,max}$) is realized when the fraction of biexcitons in the system (p_{XX}) is 1, and the fractions of single excitons (p_X) and unexcited QDs (p_0) are zero, that is, $p_{XX} = 1$ and $p_X = p_0 = 0$. In the case of an arbitrary excitation level, modal gain is linked to probabilities p_i by the following expression[3]:

$$G_{mod} = G_{mod,max}(p_{XX} - p_0)\ . \quad (2)$$

In the case of population inversion, which requires high excitation levels, p_0 is usually much smaller than p_{XX} and p_X.[4] Therefore, $G_{mod} \approx G_{mod,max}p_{XX}$. At the ASE threshold (eq. 1), $p_{XX} \approx \alpha_{loss}/G_{mod,max}$ and $p_X \approx 1 - p_{XX} = 1 - (\alpha_{loss}/G_{mod,max})$. In our BRW devices, $G_{mod,max} = 160$ cm^{-1} and $\alpha_{loss} = 16$ cm^{-1} (see main article), which yields $p_{XX} \approx 0.1$ and $p_X \approx 0.9$.

To compute the current density (j) required to achieve the ASE threshold ($j_{th,ASE}$), we calculate the overall recombination rate (K_{rec}) in the active region of our BRW device (V_{active}):

$$K_{rec} = n_{QD}V_{active}\,(p_{XX}/\tau_{XX} + p_X/\tau_X) = (f_{vol}/V_{QD})A_{inj}d_{QD}(p_{XX}/\tau_{XX} + p_X/\tau_X). \quad (3)$$

Here, τ_{XX} and τ_X are, respectively, the biexciton and single-exciton lifetimes, $n_{QD} = f_{vol}/V_{QD}$ is the QD concentration in the active region, f_{vol} is the QD volume fraction, V_{QD} is the volume of the individual QD, and $V_{active} = A_{inj}d_{QD}$, where A_{inj} is the injection area, and d_{QD} is the thickness of the active QD layer.

In the steady-state case,

$$K_{rec} = A_{inj}(j/e), \quad (4)$$

where e is the elementary charge. Combining eqs. 3 and 4, we obtain

$$j = (ef_{vol}d_{QD}/V_{QD})(p_{XX}/\tau_{XX} + p_X/\tau_X) = ef_{vol}d_{QD}/(V_{QD}\,\tau_{eff}), \quad (5)$$

where τ_{eff} is the effective electron-hole-pair lifetime in the device active volume. Here, it is defined by $\tau_{eff} = (p_{XX}/\tau_{XX} + p_X/\tau_X)^{-1}$. For the values of p_{XX} and p_X required to achieve the ASE threshold and experimentally measured biexciton and single-exciton lifetimes (1.2 ns and 12.7 ns,

respectively; Supplementary Fig. 2), $\tau_{eff} = 6.49$ ns. Using this effective lifetime and the parameters of our devices, we obtain $j_{th,ASE} = 27.6$ A cm^{-2}.

Singly negatively charged QDs. Here, we consider the case for which all QDs in the active volume are uniformly charged with a single electron. We further assume that the injection level is limited to one exciton per dot. This implies that p_{XX} is 0, and at the maximal injection current considered in the modeling, $p_X = 1$ and $p_0 = 1 - p_X = 0$. In this case, the maximal modal gain is $G_{mod,max}/2$, that is, half of the maximal biexcitonic gain.[5]

The modal gain at intermediate j can be presented as follows[5]:

$$G_{mod} = 0.5G_{mod,max}(p_X - p_0) = 0.5G_{mod,max}(2p_X - 1). \qquad (6)$$

This yields the following condition for the ASE threshold $(2p_X - 1) = 2\alpha_{loss}/G_{mod,max}$ or $p_X = \alpha_{loss}/G_{mod,max} + 1/2$, leading to $p_X = 0.6$. The corresponding threshold current density can be found from

$$j = (ef_{vol}d_{QD}/V_{QD})(p_X/\tau_{X-}) = ef_{vol}d_{QD}/(V_{QD}\tau_{eff}), \qquad (7)$$

where $\tau_{eff} = \tau_{X-}/p_X$ and τ_{X-} is the lifetime of the negatively charged exciton (negative trion). Based on our measurements (Supplementary Fig. 2), $\tau_{X-} = 4.1$ ns, which yields $\tau_{eff} = 6.8$ ns. Using this value, we obtain $j_{th,ASE} = 26.3$ A cm^{-2}.

Doubly negatively charged QDs. In the case of doubly negatively charged QDs, the maximal modal gain achievable with the injection of a single exciton is limited to $G_{mod,max}/2$, as for singly charged QDs.[5] However, since in this case unexcited QDs do not contribute to absorption, G_{mod} at intermediate injection levels is determined by

$$G_{mod} = 0.5G_{mod,max}p_X. \qquad (8)$$

At the ASE threshold, $0.5G_{mod,max}\,p_X = \alpha_{loss}$, which yields $p_X = 0.2$. The effective lifetime is then defined by $\tau_{eff} = (\tau_{X2-})/p_X$, where τ_{X2-} is the lifetime of the doubly negatively charged exciton. Using $\tau_{X2-} = 2.4$ ns (Supplementary Fig. 2) and the derived value of p_X, we obtain $\tau_{eff} = 12$ ns. This yields $j_{th,ASE} = 14.9$ A cm^{-2}.

The conducted analysis indicates that the increase in the degree of QD charging leads to the progressive reduction of the ASE threshold, which in our case, varies from ~28 A cm^{-2} for the neutral QDs to ~15 A cm^{-2} for the doubly negatively charged QDs.

Supplementary Figures

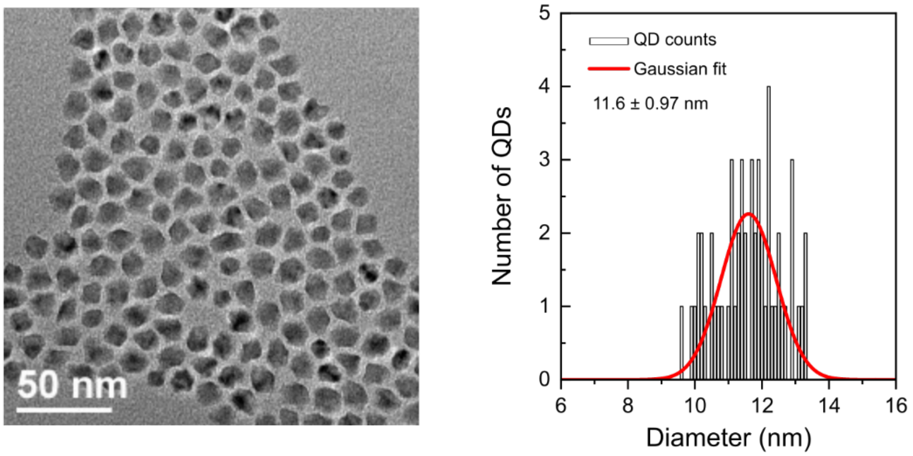

Supplementary Figure 1. Size characterization of a representative sample of $CdSe/Cd_{1-x}Zn_xSe/ZnSe_{0.5}S_{0.5}/ZnS$ compact continuously graded quantum dots (ccg-QD). Transmission electron microscopy (TEM) image of the ccg-QDs (left) and the analysis of their size distribution. The mean ccg-QD size is 11.6 nm (overall diameter) and the standard deviation is 0.97 nm.

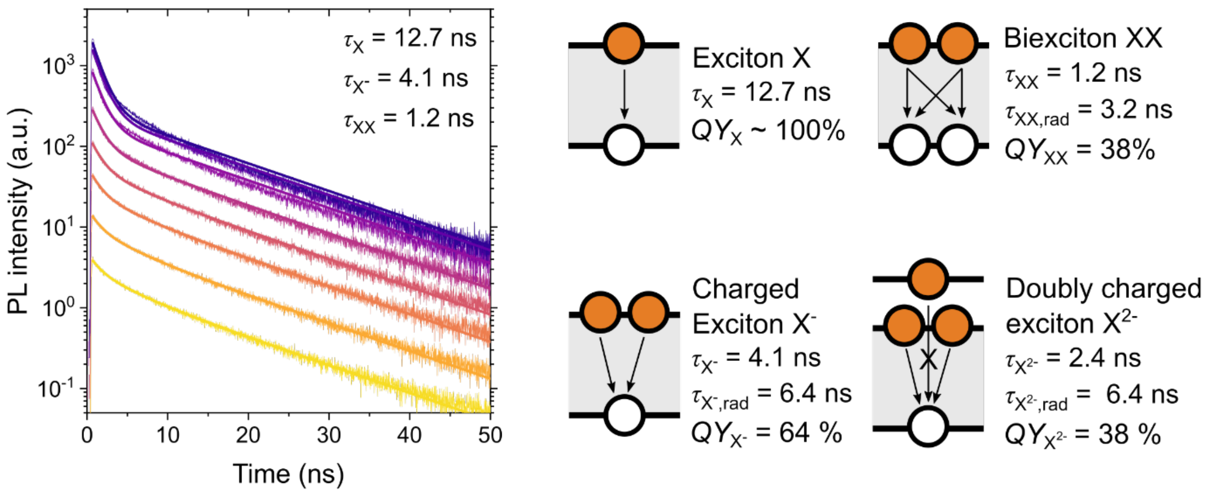

Supplementary Figure 2. Photoluminescence (PL) dynamics and emission quantum yields (QYs) of various electronic states of the ccg-QDs. (Left) Pump-intensity-dependent PL dynamics obtained using excitation with 40-fs, 400-nm (3.1-eV) laser pulses. The single exciton (τ_X = 12.7 ns), singly negatively charged exciton (τ_{X^-} = 4.1 ns), and biexciton (τ_{XX} = 1.2 ns) lifetimes are inferred from a global three-exponential fit. (Right) A schematic depiction of radiative pathways (arrows) for single-exciton (X), biexciton (XX), singly (X^-) and doubly (X^{2-}) negatively charged excitons with corresponding lifetimes and PL QYs. Radiative lifetimes are obtained using a standard 'statistical' scaling: $\tau_{XX,rad} = \tau_{X,rad}/4 \approx \tau_X/4 = 3.2$ ns, $\tau_{X^-,rad} = \tau_X/2 = 6.4$ ns. The biexciton Auger lifetime ($\tau_{XX,A}$ = 1.9 ns) is obtained from $(\tau_{XX,A})^{-1} = (\tau_{XX})^{-1} - (\tau_{XX,rad})^{-1}$.[6] The Auger recombination rate of the singly charged exciton is calculated using $(\tau_{X^-,A})^{-1} = (\tau_{X^-})^{-1} - (\tau_{X^-,rad})^{-1}$, which yields $\tau_{X^-,A}$ = 11.5 ns. The Auger lifetime of the doubly-charged exciton ($\tau_{X^{2-},A}$) is calculated based on $\tau_{X^-,A}$ with the help of the Auger decay rate scaling $r_A = (\tau_A)^{-1} = (\tau_{X^-,A})^{-1} N_e N_h (N_e + N_h - 2)/2$ (ref.[6]). This yields $\tau_{X^{2-},A}$ = 3.8 ns. The PL QY of a specific state is computed from QY = τ/τ_{rad}, where τ and τ_{rad} are the overall and the radiative lifetime of this state, respectively.

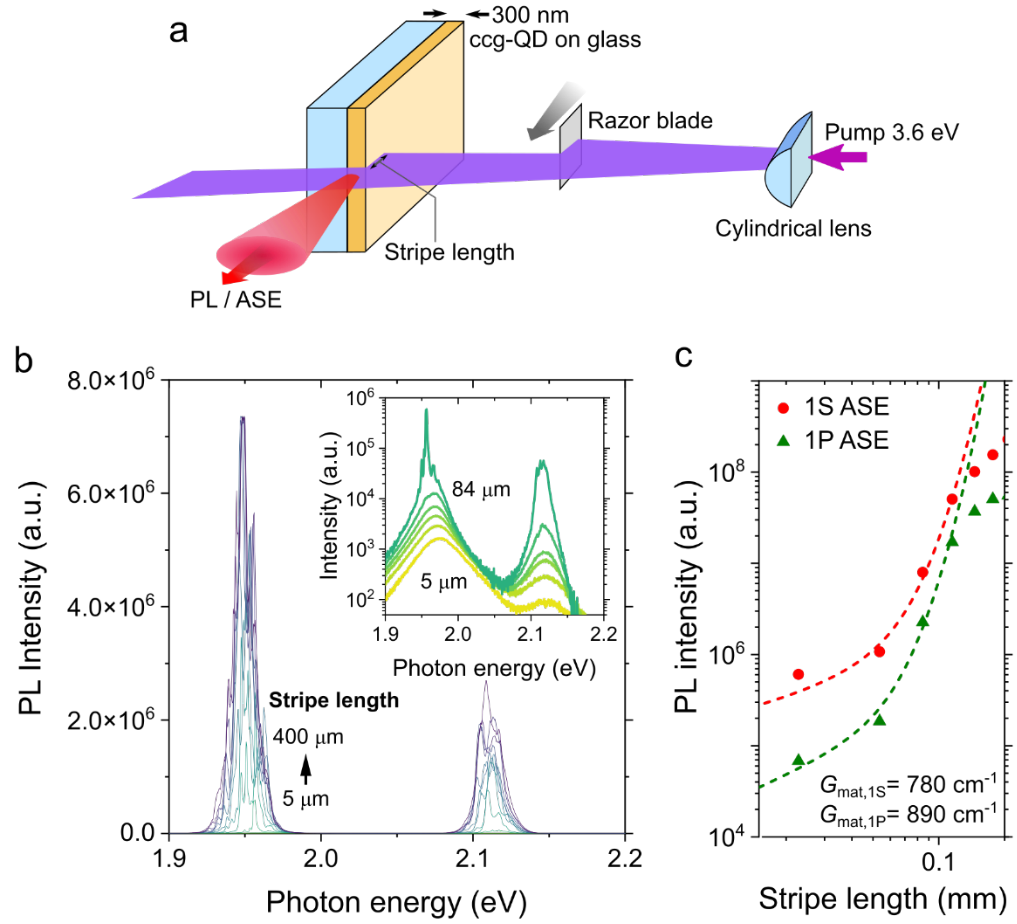

Supplementary Figure 3. Variable stripe length (VSL) measurements of the ccg-QD film prepared on a glass substrate. a, The ccg-QD film (300 nm thickness) is excited at 3.6 eV with 110-fs pulses with a per-pulse fluence which corresponds to excitation of $\langle N \rangle = 12$ excitons per dot on average. The pump beam is focused with a cylindrical lens onto the sample into a narrow stripe which is perpendicular to the sample edge. The stripe length is varied from $l = 5$ μm to 400 μm using a razor blade translated in the direction perpendicular to the pump beam (top). **b,** The edge-emitted PL spectra as a function of l (bottom). The inset shows a series of PL spectra for shorter l (≤ 84 μm) that exhibit the transition from spontaneous emission to ASE followed by the emergence of sharp features due to random lasing. **c,** The dependence of the 1S and 1P ASE signals on stripe length. The fits (dashed lines) yield optical gain coefficients 780 cm^{-1} and 890 cm^{-1} for the 1S and 1P transitions, respectively. The mode confinement for the 300-nm-thick ccg-QD film is close to 1. Therefore, we will refer to the derived gain coefficients as 'material' 1S and 1P gain (denoted in the main article as $G_{mat,1S}$ and $G_{mat,1P}$, respectively).

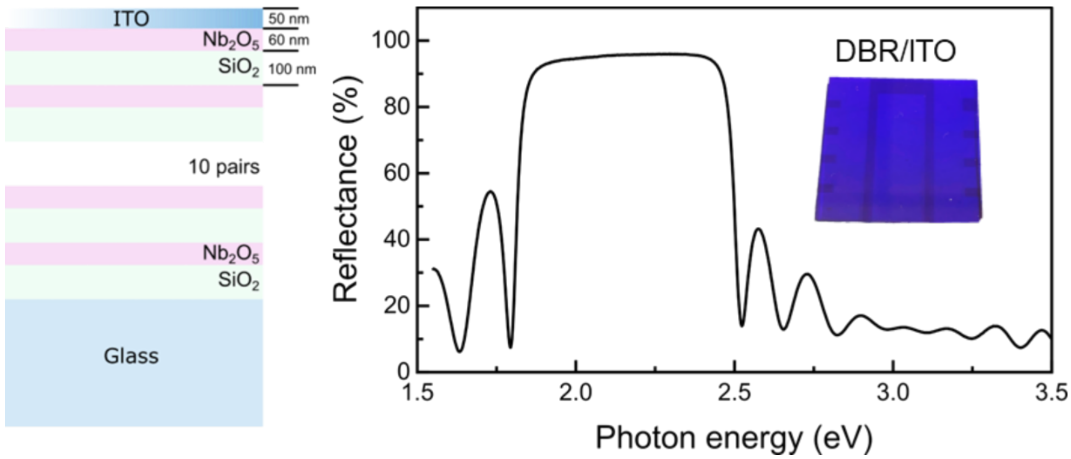

Supplementary Figure 4. An ITO-coated distributed Bragg reflector (DBR) used as a substrate for the preparation of the BRW devices. (Left) A cross-sectional structure of the ITO/DBR substrate. It comprises ten pairs of Nb_2O_5/SiO_2 layers (60 and 100 nm thicknesses, respectively) deposited on top of a glass plate. The thickness of the ITO layer is 50 nm. (Right) The measured reflectance spectrum of the DBR/ITO substrate, shows >95% reflectance within a stopband ranging from 1.77 eV to 2.48 eV. Inset: a photograph of the DBR/ITO substrate with a contact grid.

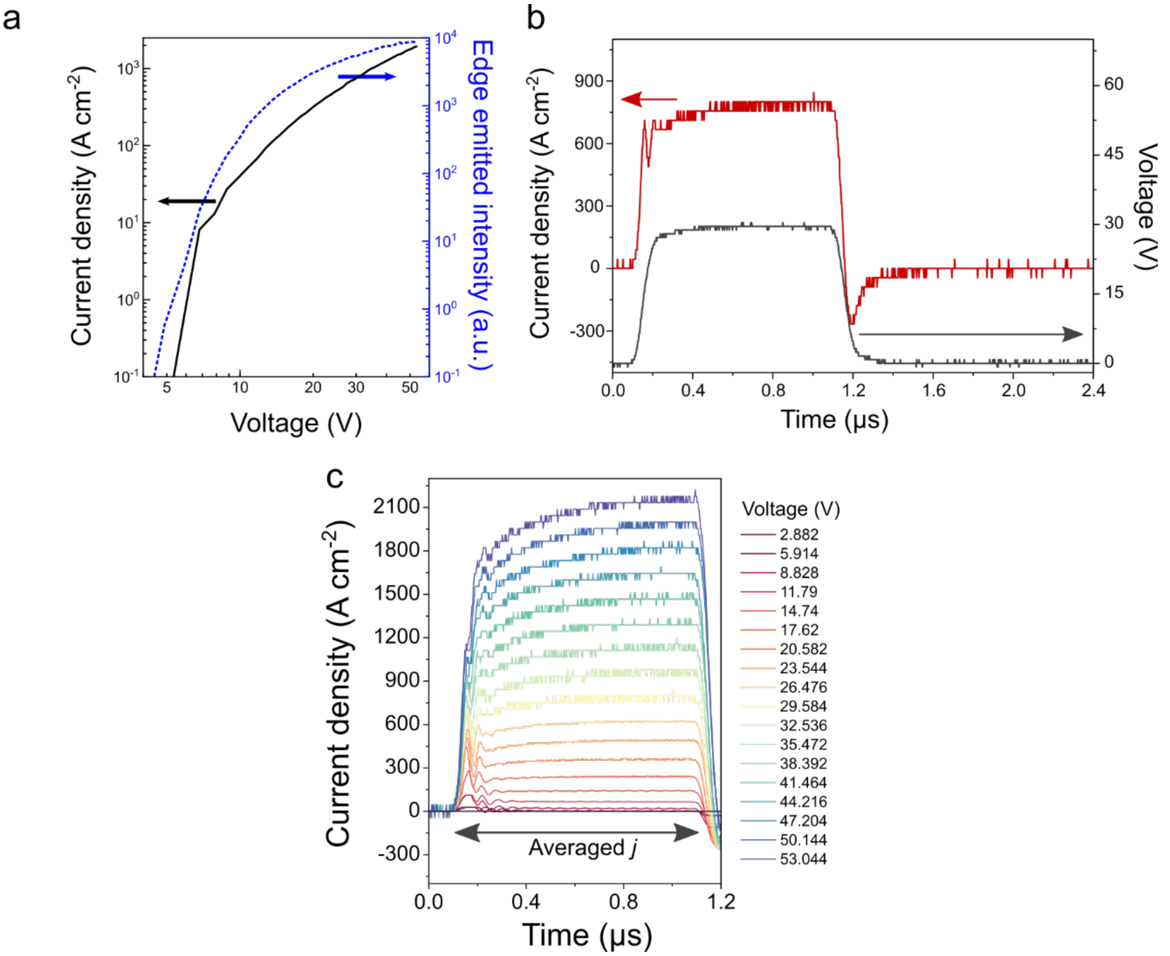

Supplementary Figure 5. Electrical characteristics of the BRW device. a, A *j*-*V* (black) and light-intensity-*V* (blue) characteristics of the edge-emitting BRW device under pulsed bias (1-μs pulse duration, 1-kHz repetition rate). The values of *j* and *V* are obtained by averaging the current density and voltage profiles (shown in panels **b** and **c**) over the 1-μs voltage pulse. **b,** An example of transient profiles of the applied voltage (black, right axis) and the current density (red, left axis). **c,** Current density profiles as a function of voltage-pulse amplitude varied from ~2.9 V to ~53 V.

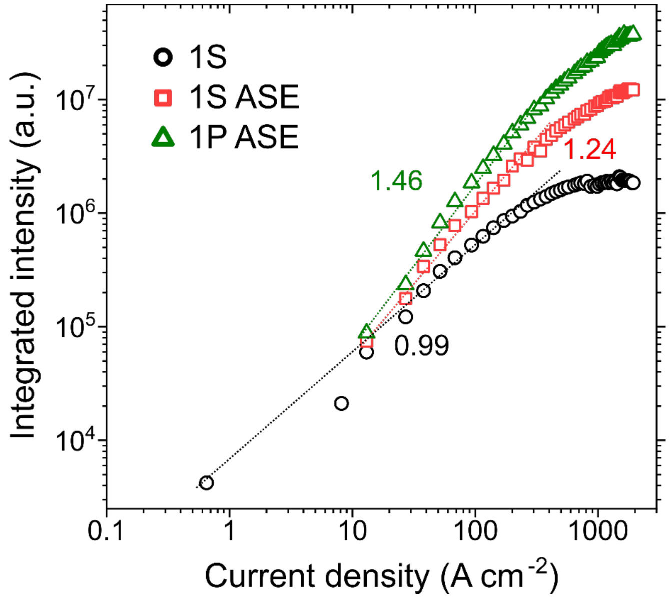

Supplementary Figure 6. Spectrally integrated electroluminescence (EL) intensities of the 1S spontaneous emission band (black), and 1S (red) and 1P (green) ASE features as a function of current density. Above j = 13 A cm^{-2}, the intensities of the 1S and 1P ASE features show super-linear growth. The corresponding log-log slopes (m = 1.24 and 1.46) are higher than that of the spontaneous EL (m = 0.99).

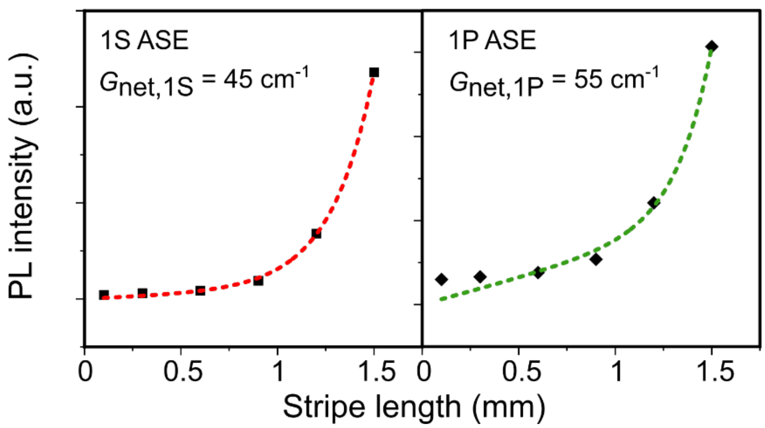

Supplementary Figure 7. VSL measurements of the BRW device (110-fs, 3.6-eV pump pulses). These measurements were used to derive net optical-gain coefficient $G_{net} = G_{mod} - a_{loss}$, where G_{mod} is modal gain and a_{loss} is the optical-loss coefficient. The plots show the edge emitted 1S (left) and 1P (right) PL intensities of the optically excited BRW device as a function of stripe length tuned from 0.1 to 1.5 mm (see Fig. 3e of the main article). Based on these measurements, the net 1S and 1P gain coefficients are 45 and 55 cm^{-1}, respectively.

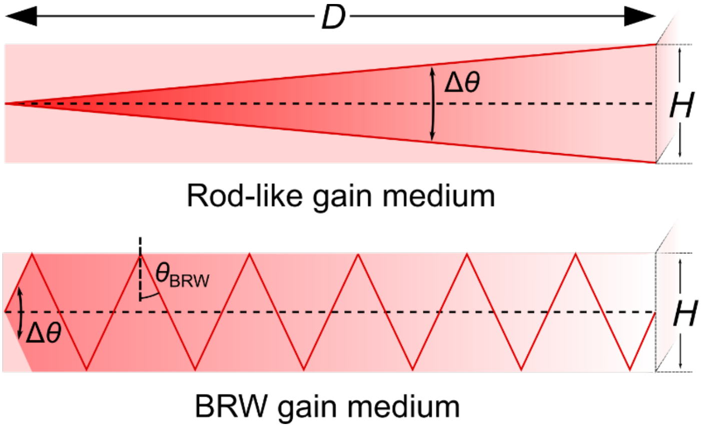

Supplementary Figure 8. Mechanisms for improved light amplification in a BRW device. An illustration of light propagation in a BRW device (bottom) versus a long rod-like light amplifier (top).[7,8] One factor underlying the improvement is the enhanced polar angle ($\Delta\theta$), which controls the fraction of spontaneous emission participating in the ASE process, and the other is a saw-like light propagation path, which increases the effective amplification length. In a mirror-less ASE device, the radiated light intensity is controlled by the effective amplification length (l_{amp}) and the fraction of overall spontaneous emission (f_{sp}) which seeds the ASE process.[7,8] For a long rod-like gain medium, l_{amp} is defined by the rod length (D), while f_{sp} can be related to the solid angle ($\Delta\Omega_{sp}$) of a small emitting volume at one end of the rod as seen from the other end ($f_{sp} \propto \Delta\Omega_{sp}/4\pi$; top).[7,8] The polar angle ($\Delta\theta$) defining $\Delta\Omega_{sp}$ can be approximated by $\Delta\theta \approx H/D$, where H is the cross-sectional size of the gain medium (top). For the dimensions of an optical-gain region in our devices, $l_{amp} = 300$ μm and $\Delta\theta \approx 2\times10^{-4}$ rad. The use of a BRW approach allows for boosting both l_{amp} and $\Delta\theta$. In particular, due to a saw-like propagation path, l_{amp} is enhanced by a factor of $(\sin\theta_{BRW})^{-1}$ (bottom), which leads to $l_{amp} = 566$ μm (computed for $\theta_{BRW} = 32^o$; Extended Data Fig. 4b). Even a greater enhancement is obtained for $\Delta\theta$. In the BRW case, this quantity is defined by $\Delta\theta = \pi - 2\theta_{BRW}$ (bottom). This yields $\Delta\theta \approx 2$ rad, which is a factor of 10^4 improvement versus the rod-like gain medium.

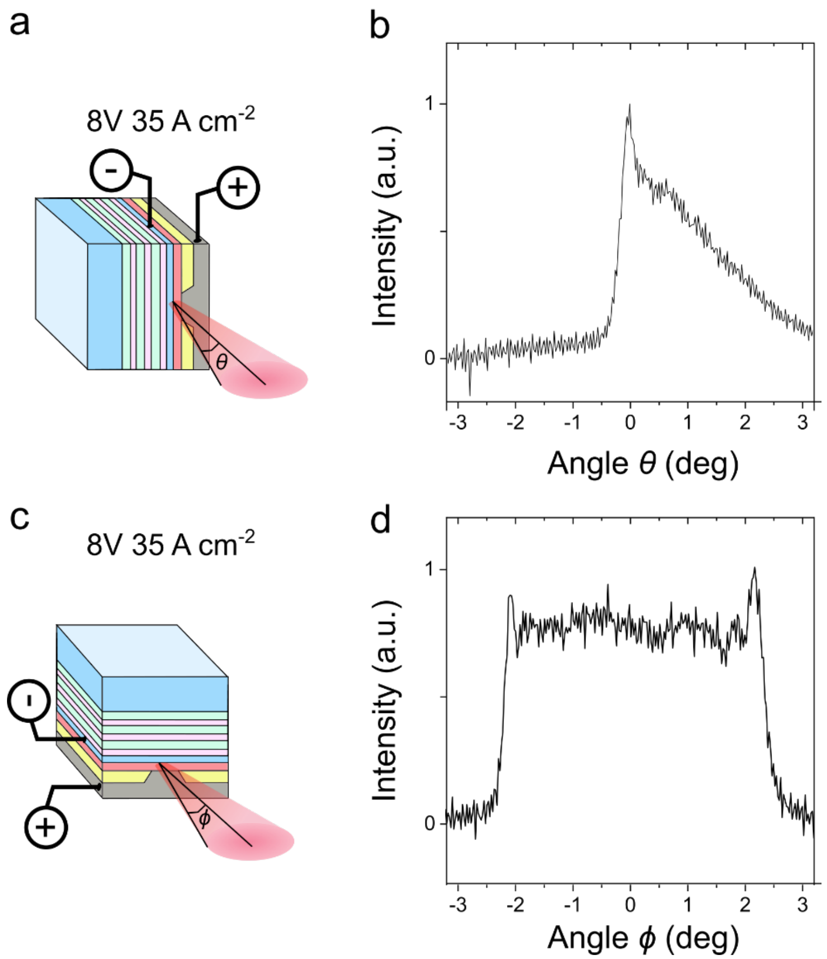

Supplementary Figure 9. Measurements of edge-emitted beam divergence of the BRW device. The measurements of beam divergence were performed using a Fourier-plane imaging setup employing a standard three-lens system and a CCD camera.[9] By varying device orientation, we obtained angular distributions of the emitted intensity in the vertical (**a,b**; angle θ) and horizontal (**c,d**; angle ϕ) directions that correspond to out-of-plane and in-plain divergence, respectively. Based on the vertical scan (**a,b**), the intensity distribution exhibits a sharp 'hot' spot with very low divergence (-0.2° to 0.2°), which is responsible for around 25 % of the total intensity. The signal falls off sharply on the silver-electrode-side of the device (negative angles) and more slowly on its DBR side (positive angles). This type of asymmetry is consistent with calculations depicted in Fig. 2b (left) of the main article that indicate considerable expansion of the BRW mode into the Bragg reflector substrate. Based on the overall beam profile, the total vertical divergence is around 2.7°. The intensity profile in the horizonal directions (**c,d**) is virtually flat within the range of the measured angles (-2.3° to 2.3°), as expected for our devices that lack any angle-selection elements in the horizontal plane. We would like to point out that in the conducted measurements the cleaved edge of the device was displaced by around 100 μm from the active device area. This might have resulted in the increased divergence versus the situation of direct emission into air.

Supplementary References

1 Ahn, N., Park, Y.-S., Livache, C., Du, J., Gungor, K., Kim, J. & Klimov, V. I. Optically Excited Lasing in a Cavity-Based, High-Current-Density Quantum Dot Electroluminescent Device. *Adv. Mater.* **35**, 2206613 (2023).

2 Roh, J., Park, Y.-S., Lim, J. & Klimov, V. I. Optically pumped colloidal-quantum-dot lasing in LED-like devices with an integrated optical cavity. *Nat. Commun.* **11**, 271 (2020).

3 Park, Y.-S., Roh, J., Diroll, B. T., Schaller, R. D. & Klimov, V. I. Colloidal quantum dot lasers. *Nat. Rev. Mater.* **6**, 382-401 (2021).

4 Jung, H., Ahn, N. & Klimov, V. I. Prospects and challenges of colloidal quantum dot laser diodes. *Nat. Photon.* **15**, 643-655 (2021).

5 Kozlov, O. V., Park, Y.-S., Roh, J., Fedin, I., Nakotte, T. & Klimov, V. I. Sub–single-exciton lasing using charged quantum dots coupled to a distributed feedback cavity. *Science* **365**, 672-675 (2019).

6 Klimov, V. I. Multicarrier Interactions in Semiconductor Nanocrystals in Relation to the Phenomena of Auger Recombination and Carrier Multiplication. *Annu. Rev. Condens. Matter Phys.* **5**, 285-316 (2014).

7 Casperson, L. W. Threshold characteristics of mirrorless lasers. *J. Appl. Phys.* **48**, 256-262 (1977).

8 Siegman, A. E. Ch. 13.8 Amplified Spontaneous Emission and Mirrorless Lasers, (University Science Books, 1986).

9 Wagner, R., Heerklotz, L., Kortenbruck, N. & Cichos, F. Back focal plane imaging spectroscopy of photonic crystals. *Appl. Phys. Lett.* **101**, 081904 (2012).
