# Supplementary material for: Electrically driven amplified spontaneous emission from colloidal quantum dots
Source: Nature. 2023 May 3;617(7959):79–85. doi: 10.1038/s41586-023-05855-6 (PMC10156592; doi:10.1038/s41586-023-05855-6)
Supplement: Supplementary file 2 — Reporting Summary [file 41586_2023_5855_MOESM2_ESM.pdf]

Corresponding author(s): Victor I. Klimov

Lasing Reporting Summary

Nature Research wishes to improve the reproducibility of the work that we publish. This form is intended for publication with all accepted papers reporting claims of lasing and provides structure for consistency and transparency in reporting. Some list items might not apply to an individual manuscript, but all fields must be completed for clarity.

For further information on Nature Research policies, including our data availability policy, see Authors & Referees.

Experimental design

Please check: are the following details reported in the manuscript?

1. Threshold

Plots of device output power versus pump power over a wide range of values indicating a clear threshold

☒ Yes ☐ No

For both optically and electrically pumped devices, the ASE thresholds are indicated in the main text. The threshold-like ASE onset is illustrated in Fig. 1c (optical excitation) and Fig. 3b,c (electrical excitation).

2. Linewidth narrowing

Plots of spectral power density for the emission at pump powers below, around, and above the lasing threshold, indicating a clear linewidth narrowing at threshold

☒ Yes ☐ No

Fig. 1c (optical excitation) and Fig. 3b,c (electrical excitation). These data indicate both the threshold behavior of the output intensity and line narrowing during the transition from spontaneous emission to ASE.

Resolution of the spectrometer used to make spectral measurements

☒ Yes ☐ No

0.1 nm

3. Coherent emission

Measurements of the coherence and/or polarization of the emission

☒ Yes ☐ No

Fig. 3d and Extended Data Fig. 7 - measurements of polarization indicate the preferred TE polarization for ASE and no preferred polarization for spontaneous emission. Extended Data Fig. 8 - temporal coherence measurements, which indicate a longer coherence time for ASE compared to spontaneous emission.

4. Beam spatial profile

Image and/or measurement of the spatial shape and profile of the emission, showing a well-defined beam above threshold

☒ Yes ☐ No

Sup Fig. 9 - beam divergence measurements.

5. Operating conditions

Description of the laser and pumping conditions
Continuous-wave, pulsed, temperature of operation

☒ Yes ☐ No

These ASE devices are excited with 1-mks voltage pulses at 1 kHz repetition rate (main text and Fig. 4 caption). This corresponds to quasi-d.c. excitation as the pulse duration is longer than the single exciton lifetime (~10 ns).

Threshold values provided as density values (e.g. W cm^{-2} or J cm^{-2}) taking into account the area of the device

☒ Yes ☐ No

ASE thresholds are provided as current density (j_th,ASE = 13 A cm^-2)

6. Alternative explanations

Reasoning as to why alternative explanations have been ruled out as responsible for the emission characteristics
e.g. amplified spontaneous, directional scattering; modification of fluorescence spectrum by the cavity

☒ Yes ☐ No

The effect claimed in this paper is ASE. We have provided a detailed analysis of alternative explanations including spectral filtering due to transverse cavity effects and spontaneous emission from multiexcitons.

7. Theoretical analysis

Theoretical analysis that ensures that the experimental values measured are realistic and reasonable
e.g. laser threshold, linewidth, cavity gain-loss, efficiency

☒ Yes ☐ No

Yes, we have conducted a quantitative analysis of modal gain and losses (COMSOL), and the ASE thresholds. The theoretical results are in excellent agreement with the experimental observations.

8. Statistics

Number of devices fabricated and tested

☒ Yes ☐ No

A total of 15 chips were fabricated. Each chip contained 8 devices. This paper is based on measurements of devices from 11 chips. Extended Data Fig. 10 - analysis of reproducibility based on the measurements of 11 devices.

Statistical analysis of the device performance and lifetime (time to failure)

☒ Yes
☐ No

Extended Data Fig. 9 - studies of operational stability. Stable ASE regime is maintained for several hours of operation.
